# Global Perceptions on ERAS^®^ in Pancreatoduodenectomy

**DOI:** 10.1007/s00268-023-07198-9

**Published:** 2023-10-03

**Authors:** Monish Karunakaran, Didier Roulin, Shahid Ullah, Shailesh V. Shrikhande, Hans D. De Boer, Nicolas Demartines, Savio George Barreto

**Affiliations:** 1 Department of Surgical Gastroenterology, Asian Institute of Gastroenterology, Hyderabad, 500 032 India; 2 College of Medicine and Public Health, Flinders University, Bedford Park, South Australia Australia; 3 Department of Visceral Surgery, Lausanne University Hospital CHUV and University of Lausanne UNIL, 1011 Lausanne, Switzerland; 4 Department of Gastrointestinal and HPB Surgical Oncology, Tata Memorial Hospital, Mumbai, India; 5 Homi Bhabha National Institute, Training School Complex, Anushakti Nagar, Mumbai, 400085 India; 6 Department of Anesthesiology, Pain Medicine and Procedural Sedation and Analgesia, Martini General Hospital Groningen, Groningen, The Netherlands; 7 Division of Surgery and Perioperative Medicine, Flinders Medical Center, Bedford Park, Adelaide, South Australia 5042 Australia

## Abstract

**Background:**

Uptake of ERAS^®^ pathways for pancreatic surgery have been slow and impacted by low compliance.

**Objective:**

To explore global awareness, perceptions and practice of ERAS^®^ peri-pancreatoduodenectomy (PD).

**Methods:**

A structured, web-based survey (EPSILON) was administered through the ERAS^®^ society and IHPBA membership.

**Results:**

The 140 respondents included predominantly males (86.4%), from Europe (45%), practicing surgery (95%) at academic/teaching hospitals (63.6%) over a period of 10–20 years (38.6%). Most respondents identified themselves as general surgeons (68.6%) with 40.7% reporting an annual PD volume of 20–50 cases, practicing post-PD clinical pathways (37.9%), with 31.4% of respondents auditing their outcomes annually. Reduced medical complications, cost and hospital length of stay, and improved patient satisfaction were perceived benefits of compliance to enhancing-recovery. Multidisciplinary co-ordination was considered the most important factor in the implementation and sustainability of peri-PD ERAS^®^ pathways, while reluctance to change among health care practitioners, difficulties in data collection and audit, lack of administrative support, and recruitment of an ERAS^®^ dedicated nurse were reported to be important barriers.

**Conclusions:**

The EPSILON survey highlighted global clinician perceptions regarding the benefits of compliance to peri-PD ERAS^®^, the importance of individual components, perceived facilitators and barriers, to the implementation and sustainability of these pathways.

**Supplementary Information:**

The online version contains supplementary material available at 10.1007/s00268-023-07198-9.

## Introduction

The terminology and conceptual framework for accelerating and improving postoperative recovery using a standardized and multidisciplinary perioperative pathway, “enhanced recovery after surgery” (ERAS) was introduced by a group of academic colorectal surgeons [1, 2]. Following the publication of the first ERAS^®^ guidelines for colorectal surgery in 2005 [3], an appreciation of the beneficial experiences with similar clinical pathway programmes following pancreatoduodenectomy (PD) led to the first comprehensive consensus framework for optimal perioperative care after PD [4], which were later updated in 2020 [5].

Despite the availability of evidence that ERAS^®^ pathways for PD reduce post-operative length of stay [6], overall morbidity [7] and costs [8] without an increase in major complications, readmissions, reoperations, or mortality[6], its uptake has been slow in pancreatic surgery and has been dogged by low compliance even in centers that have adopted the pathways to enhance recovery [9]. While it may be surmised that availability of resources, or lack thereof, and the reluctance to change individual practice guided by dogmatism that focuses on the lack of sufficient evidence [10] of the benefits of ERAS^®^, may be some of the reasons, the fact remains that global perceptions of ERAS^®^ in PD have not been systematically explored.

The objective of the present study was to explore global awareness, perceptions and practice of ERAS^®^ in the peri-operative care of patients undergoing PD to determine facilitators and impediments to their implementation and sustainability. Such information is invaluable in terms of providing the ERAS^®^ Society with a specific agenda to be addressed including the development of educational resources, providing incentive for quality improvement strategies, and future research with the overarching aim of improving the care of patients undergoing PD.

## Methods

A structured, web-based (Qualtrics^®^) survey (entitled ‘EPSILON’—ERAS^®^ in Pancreatoduodenectomy—An International Online Study; Annexure) consisting of 20 multiple choice questions was designed and administered to clinicians involved in the care of patients undergoing PD. The survey included questions to clarify the respondents’ personal demographic, specialty, region and type of practice, and personal experience with PD, strategies at enhancing recovery. The respondents were then questioned on their perceptions relating to the benefits of specific aspects of ERAS^®^, including the components from the published guidelines [5] and perceived facilitators, challenges- and barriers to their implementation.

Support for the dissemination of the survey was engendered from the ERAS ^®^ society and the International Hepato-Pancreato-Biliary Association (IHPBA). These organizations distributed the survey to their extensive global memberships. To facilitate global catchment, the e-surveys were made available via Twitter^®^, too. Response to the survey was voluntary and anonymous. The definitions of post-pancreatectomy complications used in the survey were based on the definitions of the International Study Group of Pancreatic surgery (ISGPS), including post-operative pancreatic fistula (POPF), delayed gastric emptying (DGE), post-pancreatectomy haemorrhage (PPH), and post-pancreatectomy acute pancreatitis (PPAP) [11–14]. The items and domains relating to ERAS^®^ protocols were defined as the ERAS^®^ guidelines of 2019 [5]. In brief, there are 64 items from five domains: (i) perceptions on benefits of compliance to peri-PD ERAS^®^ (8 items); (ii) perceptions on importance of peri-PD ERAS^®^ (22 items); (iii) challenges to the application of ERAS^®^ (22 items); (iv) facilitators to implementation and sustainability of peri-PD ERAS^®^ (5 items) and (v) barriers to implementation and sustainability of peri-PD ERAS^®^ (7 items) rated on a 10-point Likert scale with ranges from not important (0) to very important (10).

## Statistical analysis

All analyses were performed using R software version 4.2. Participants’ characteristics were reported as percentages of the respective denominator. The respondent’s perceptions relating to the benefits and importance of specific aspects of ERAS^®^, and perceived facilitators, challenges- and barriers were reported as percentages of their ratings grouped as 0–4, 5, and 6–10 by using Likert plot. The respondent’s perceptions were also presented by median (25^th^–7^th^ percentiles) scores. A Mann–Whitney U and Kruskal–Wallis H tests were performed to explore the significance differences of each specific aspects of ERAS and individual perceived facilitators, challenges- and barriers by participant’s characteristics. Radar plot was used to examine the average score for specific aspects of ERAS^®^ in various domains. The two-sided test was performed for all analyses and the level of significance was set at 0.05.

## Results

### Demographics, clinical experience and peri-PD care practices (Table 1)

**Table 1:** Participant demographic and professional practice information (including pancreatic surgery and peri-operative practice)

Characteristic	N = 140
*Sex*	
Male	121 (86.4%)
Female	19 (13.6%)
*Geographical area*	
Europe	63 (45.0%)
Asia/Oceania	42 (30.0%)
North America	24 (17.1%)
South America	11 (7.9%)
*Type of institution*	
Academic/teaching hospital	89 (63.6%)
Tertiary hospital	29 (20.7%)
Private/corporate hospital	12 (8.6%)
Public/Government hospital	7 (5.0%)
Regional hospital	2 (1.4%)
Community hospital	1 (0.7%)
*Years in practice*	
<10 Years	50 (35.7%)
10–20 Years	54 (38.6%)
>20 Years	36 (25.7%)
*Speciality*	
Surgery	133 (95.0%)
Anaesthesia	5 (3.6%)
Gastroenterology	1 (0.7%)
Nursing	1 (0.7%)
*Case-Mix*	
HPB surgery	96 (68.6%)
General surgery	25 (17.9%)
Pancreatic surgery	11 (7.9%)
Others	8 (5.7%)
*Annual PD volume*	
<20	33 (23.6%)
20–50	57 (40.7%)
>50	50 (35.7%)
*Unit’s PD patient care routine practice*	
ERAS^®^ pathways	45 (32.1%)
Post-PD CP to enhance recovery	53 (37.9%)
Management at surgeon’s discretion	42 (30.0%)
*Frequency of audits*	
Never	27 (19.3%)
Monthly	22 (15.7%)
Quarterly	25 (17.9%)
Bi-Annually	17 (12.1%)
Annually	44 (31.4%)
Others	5 (3.6%)

*PD* pancreatoduodenectomy, *HPB* hepato-pancreato-biliary, *CP* clinical pathway; *ERAS*^®^ enhanced-recovery after surgery

Of the 140 respondents to the survey, 86.4% (n = 121) were male. The majority of respondents were from Europe (45%), practicing surgery (95%) at academic/teaching hospitals (63.6%) over a period of 10–20 years (38.6%). Most respondents identified themselves as general surgeons (68.6%) with 40.7% reporting an annual PD volume of 20–50 cases. The most commonly practiced form of enhancing recovery peri-PD amongst respondents were post-PD clinical pathways (37.9%) with nearly 31.4% of respondents volunteering that they audited their outcomes annually.

### Perceptions on benefits of compliance to peri-PD ERAS^®^

**Fig. 1:**
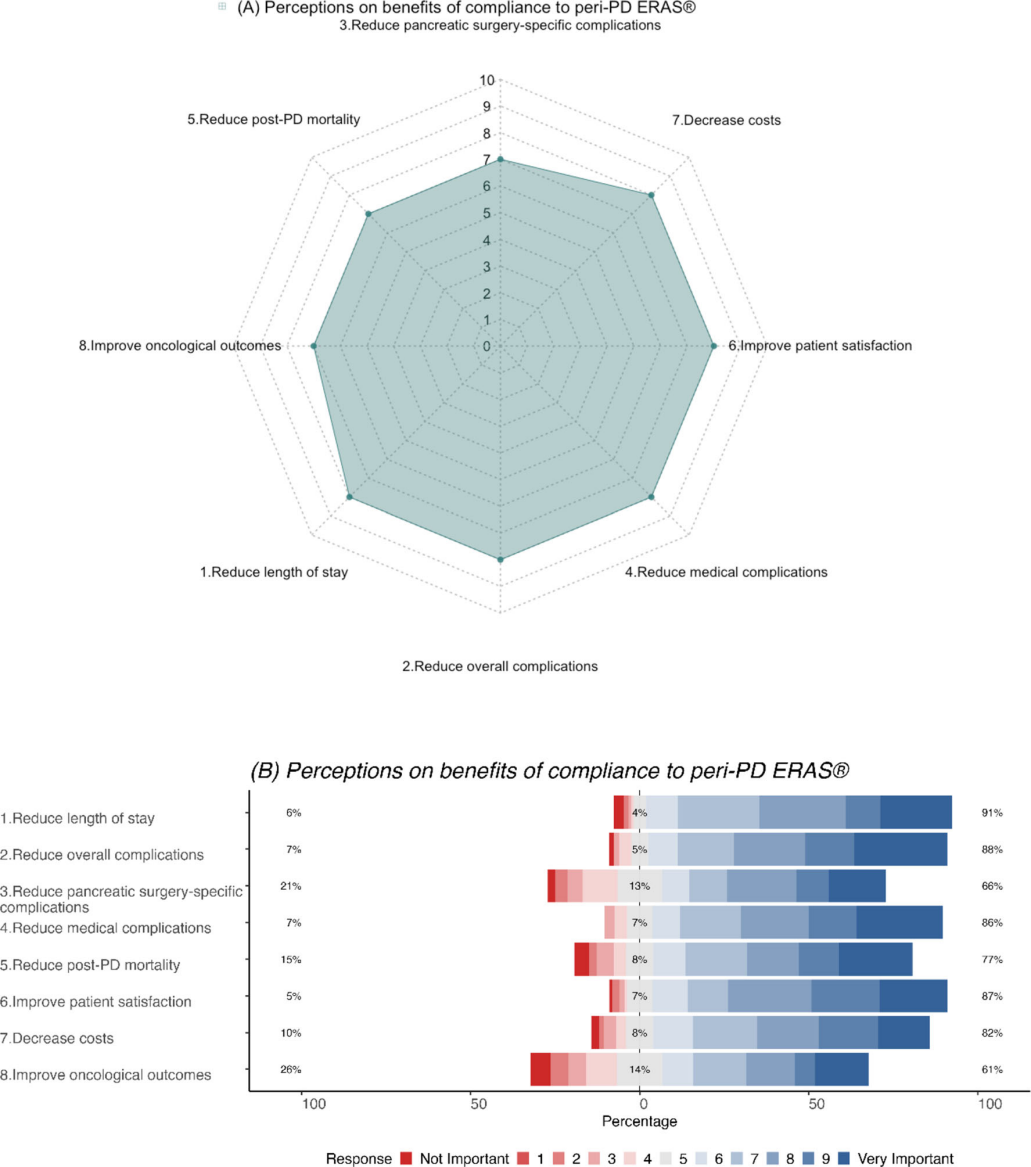
**a** Radar plot (median score) and **b** Likert plot (%) demonstrating perceptions on benefits of compliance to peri-PD ERAS^®^

The benefits of compliance to peri-PD ERAS^®^ pathways perceived to be most important amongst respondents included a reduced length of post-PD hospital stay**,** reduced overall, and medical complications, improved patient satisfaction, and reduced overall cost (median score = 8) (Fig. 1a). In particular, the majority of respondents strongly felt that compliance to ERAS^®^ protocols results in a reduced length of post-PD hospital stay (91%) overall (88%) and medical (86%) complications, and post-PD mortality (77%). They also strongly rated that compliance improved patient satisfaction (87%) and reduced overall costs (82%). However, the rates were relatively lower for pancreatic surgery-specific complications (66%) and oncological outcomes (61%) (Fig. 1b).

The above perceptions did not vary depending on the respondent’s sex, geographical location, years of practice, case-mix, PD volumes, or their peri-PD practices to enhance recovery (Supplementary Tables 1A to F).

### Perceived importance of individual components of ERAS^®^ pathways

**Fig. 2:**
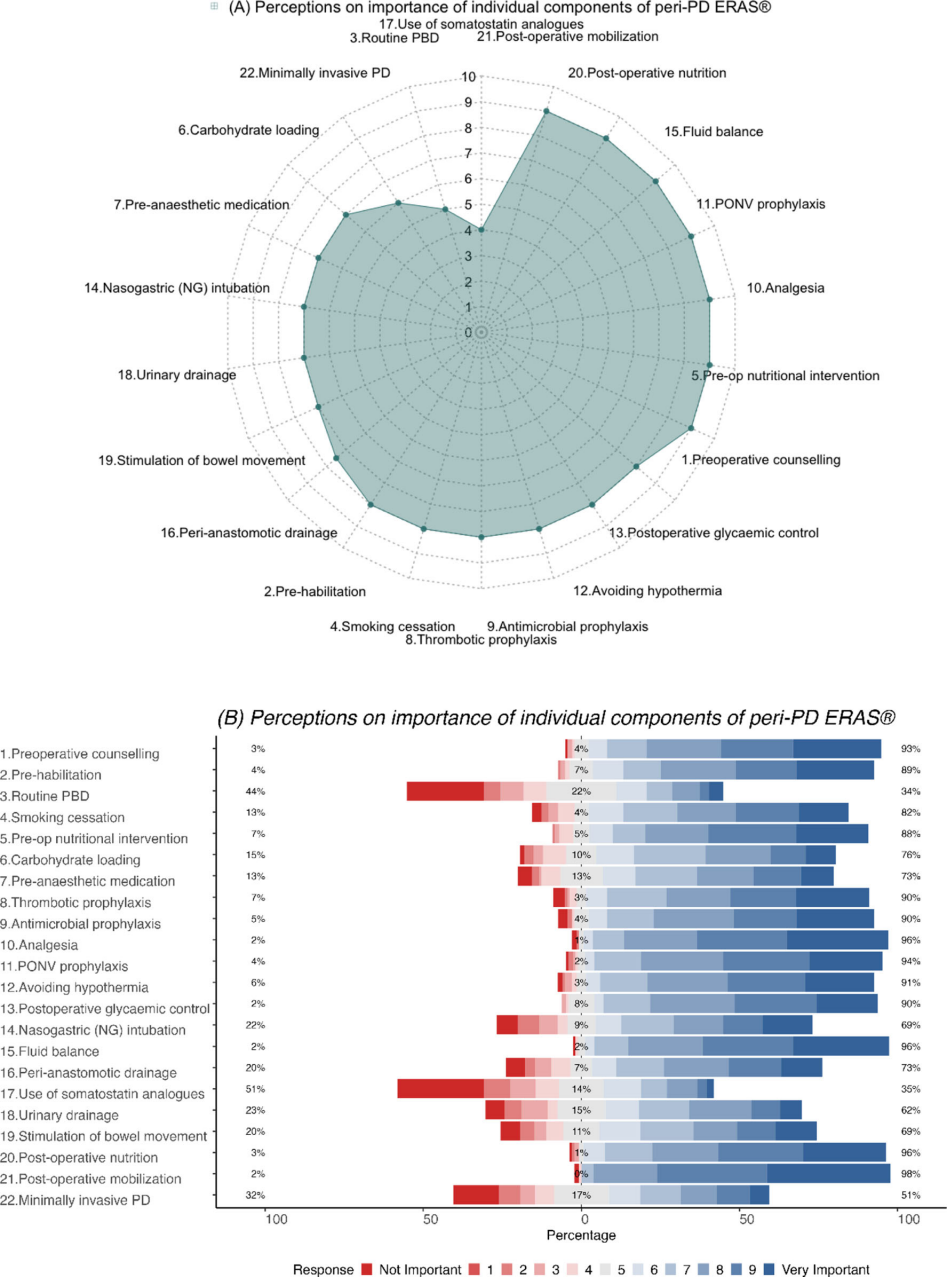
**a** Radar plot (median score) and **b** Likert plot (%) demonstrating participant perceptions on importance of individual components of peri-PD ERAS^®^

Among the individual components of ERAS^®^ pathways in PD, preoperative counselling, pre-operative nutritional intervention, post-operative analgesia, post-operative nausea and vomiting (PONV) prophylaxis, the maintenance of fluid balance, postoperative nutrition and post-operative mobilization were considered to be the most important (median score = 9), while the use of somatostatin analogues and routine pre-operative biliary drainage (PBD) were considered the least important (median scores of 4 and 5, respectively) by the respondents (Fig. 2a).

Majority of respondents rated highly (score ≥6) the importance of the following components of peri-PD ERAS^®^, namely, pre-operative counselling (93%), pre-habilitation (89%), smoking cessation (82%), preoperative nutritional intervention (88%), thrombotic prophylaxis (90%), antimicrobial prophylaxis (90%), analgesia (96%), PONV prophylaxis (94%), avoiding hypothermia (91%), postoperative glycaemic control (90%), the maintenance of fluid balance (95%), perianastomotic drainage (73%), postoperative nutrition (96%), and postoperative mobilisation (98%) (Fig. 2b).

Preoperative nutritional interventions (*p* = 0.039) and post-operative urinary drainage (*p* = 0.048) were considered significantly more important by female respondents in comparison to males (Supplementary Table 2A). Smoking cessation was reported to be significantly more important by respondents from Asia/Oceania and South America (*p* < 0.001). In addition, post-operative analgesia (*p* = 0.007) and nutrition (*p* = 0.009) were perceived to be significantly important among South American respondents (Supplementary Table 2B). Smoking cessation (*p* = 0.036), thromboprophylaxis (*p* = 0.005), PONV prophylaxis (*p* = 0.042) and post-operative mobilization (*p* = 0.049) were rated as significantly more important by respondents with >20 years of experience (Supplementary Table 2C). Respondents with an annual PD volume <20 PDs/year attributed greater importance to smoking cessation (*p* = 0.023), preoperative nutritional interventions (*p* = 0.007), postoperative analgesia (*p* = 0.005), avoidance of hypothermia (*p* = 0.005), postoperative glycaemic control (*p* = 0.001), stimulation of bowel movements (*p* = 0.003) and post-operative mobilization (*p* = 0.015) (Supplementary Table 2E). Pancreatic surgeons (*p* = 0.007) attached significantly less importance to component on minimally-invasive PD (MIPD) (Supplementary Table 2D) while it was perceived to be important by respondents employing ERAS^®^ protocols in their routine post-PD patient care (*p* = 0.019) (Supplementary Table 2F).

### Perceived challenges to the application of individual components of ERAS^®^ pathways

**Fig. 3:**
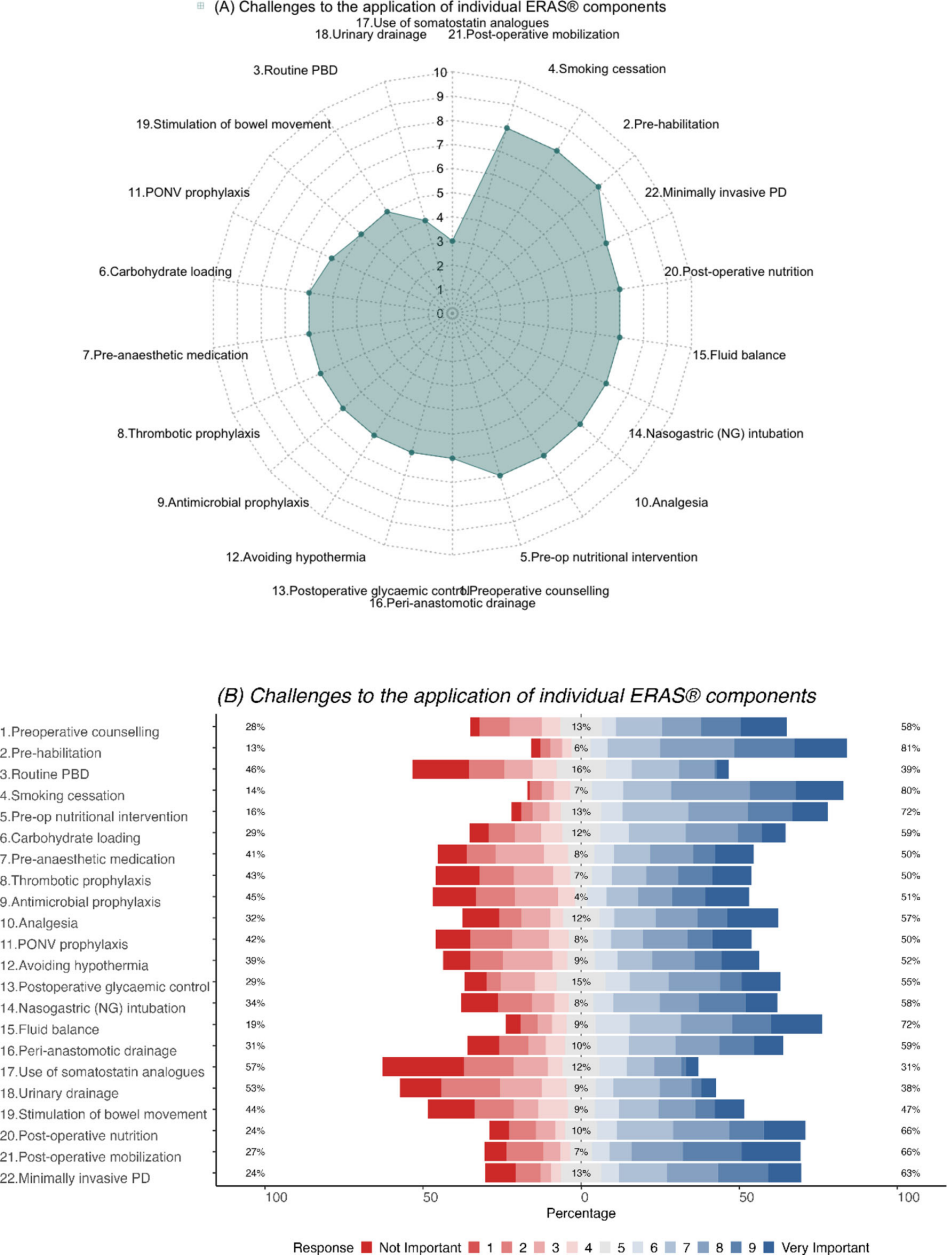
**a** Radar plot (median score) and **b** Likert plot (%) demonstrating participant perceptions on challenges to the application of individual ERAS^®^ components

The achievement of post-operative mobilization, smoking cessation and pre-habilitation were deemed to be the most challenging (median score = 8) components in ERAS^®^ (Fig. 3a). The majority of respondents strongly rated the following individual components as challenging to implement in a peri-PD setting, namely, pre-habilitation (81%), smoking cessation (80%), and pre-operative nutritional intervention (72%) (Fig. 3b). These were followed by MIPD, post-op nutrition, fluid balance, nasogastric intubation, analgesia, preoperative nutritional interventions and pre-operative counselling (median score = 7). In contrast, the use of somatostatin analogues and urinary drainage were reported to be the least challenging ones to implement (median score 3 and 4, respectively).

Female respondents (*p* = 0.047) and those from institutions utilizing ERAS^®^ pathways (*p* = 0.019) reported recommendations on MIPD difficult to implement. Respondents from Asia/Oceania found pre-operative counselling (*p* = 0.033), carbohydrate loading (*p* = 0.013), antimicrobial prophylaxis (*p* = 0.026), nasogastric intubation (*p* = 0.012), fluid balance (*p* = 0.024) and post-operative nutrition (*p* = 0.045) challenging to implement compared to other regions.

While there was no difference with respect to years in practice, or case-mix, respondents with an annual PD volume <20 PDs/year were likely to encounter significantly greater challenges in implementing smoking cessation (*p* = 0.023), pre-operative nutritional intervention (*p* = 0.007), analgesia (*p* = 0.005), avoidance of hypothermia (*p* = 0.005), postoperative glycaemic control (*p* = 0.001), stimulation of bowel movement (*p* = 0.03) and post-operative mobilization (*p* = 0.015) (Supplementary Tables 3 A–F).

### Facilitators to implementation and sustainability of peri-PD ERAS^®^ pathways

**Fig. 4:**
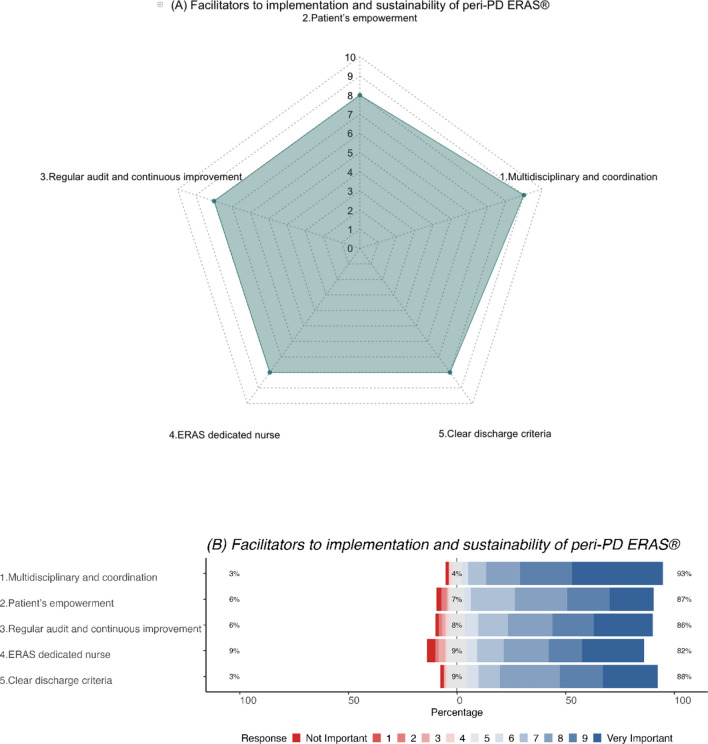
**a** Radar plot (median score) and **b** Likert plot (%) demonstrating participant perceptions on facilitators to implementation and sustainability of peri-PD ERAS^®^ pathways

Multidisciplinary co-ordination (Median score = 9) was believed to be the most important factor in the implementation and sustainability of peri-PD ERAS^®^ pathways. Other factors, namely, patient empowerment, regular audit and continuous improvement process, a dedicated ERAS^®^ nurse and clear discharge criteria were perceived to be important as well, with a median score of 8 (Fig. 4a, b).

The majority of respondents strongly rated the importance of the following facilitators to the implementation and sustainability of pathways aimed at enhancing recovery peri-PD, namely, a multidisciplinary approach with co-ordination between different members (93%), patient empowerment (87%), regular audit and continuous improvement processes (86%), dedicated ERAS nurse (82%), and clear discharge criteria (88%).

Female respondents (*p* = 0.011), and those from institutions where perioperative care was at the discretion of the individual surgeon (*p* = 0.003), believed that having clear discharge criteria was very important in peri-PD ERAS^®^ pathways (*p* = 0.011). Surgeons with >20 years of experience were of the opinion that multidisciplinary co-ordination was important in implementation and sustainability of peri-PD ERAS^®^ pathways (*p* = 0.03). When stratified by annual PD volumes, respondents performing <20 PDs/year, attached greatest importance to multidisciplinary co-ordination (*p* = 0.008), patient empowerment (*p* = 0.043), regular audit (*p* = 0.008), dedicated ERAS nurse (*p* = 0.003) and clear discharge criteria (*p* < 0.001).

While there was no significant difference with respect to case-mix, multidisciplinary co-ordination (*p* = 0.030), ERAS dedicated nurse (*p* = 0.013) and clear discharge criteria (*p* = 0.002) were believed to be significantly more important by South American respondents. However, patient’s empowerment (*p* = 0.039) was believed to be significantly more important in Asia/Oceania for implementation and sustainability of peri-PD ERAS^**®**^ pathways (Supplementary Tables 4A–F).

### Perceived barriers to implementation and sustainability of peri-PD ERAS^®^ pathways

**Fig. 5:**
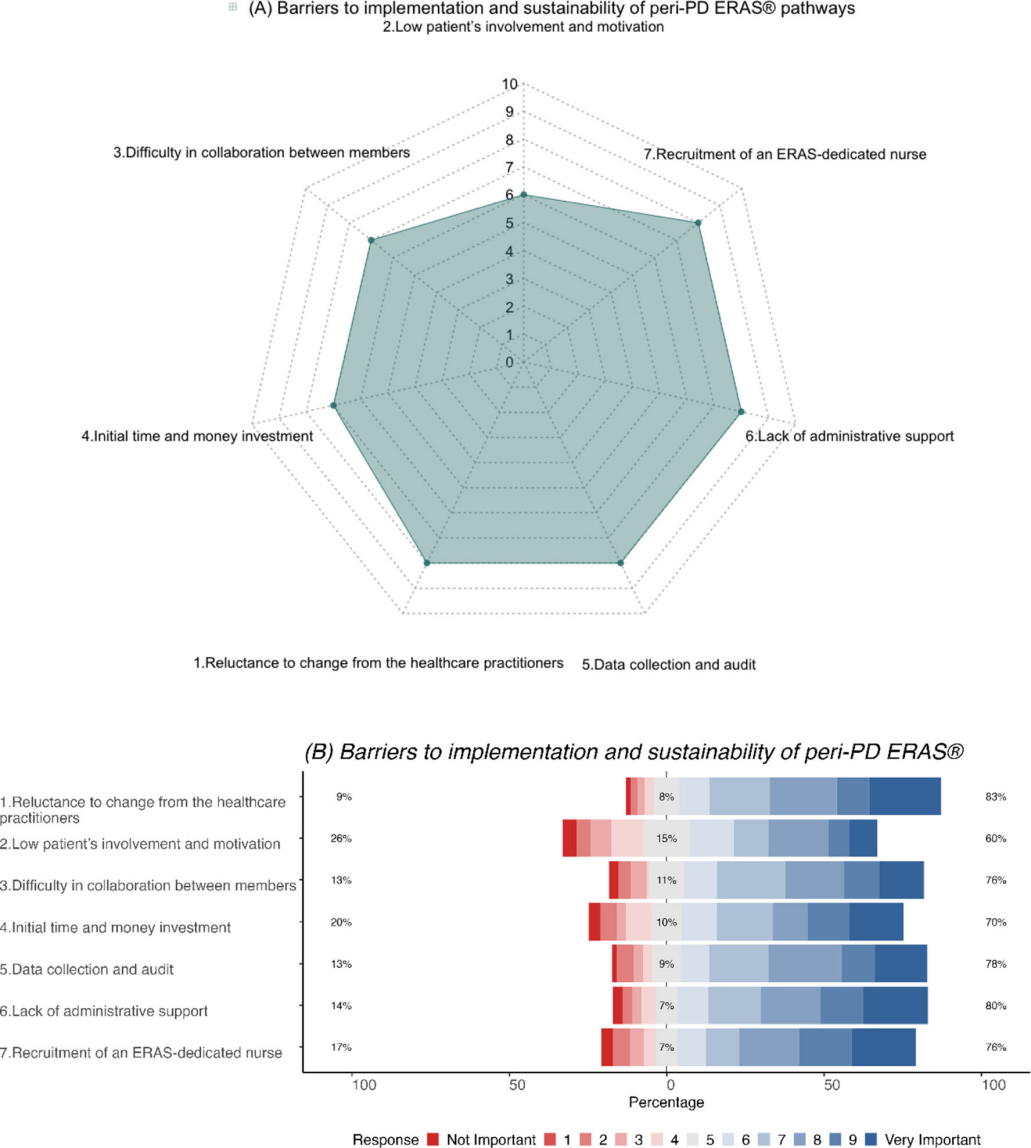
**a** Radar plot (median score) and **b** Likert plot (%) demonstrating participant perceptions on barriers to implementation and sustainability of peri-PD ERAS^®^ pathways

Reluctance to change among health care practitioners, difficulties in data collection and audit, lack of administrative support and recruitment of an ERAS^®^ dedicated nurse (median score = 8) were reported to be important barriers to the successful implementation and sustainability of peri-PD ERAS^®^ pathways (Fig. 5a, b).

Among respondents, majority strongly rated (median score ≥6) the following as important barriers to the implementation and sustainability of peri-PD ERAS^®^ pathways, namely, reluctance to change amongst healthcare practitioners (83%), difficulty in collaboration between members of the multidisciplinary teams (76%), data collection and audit (78%), lack of administrative support (80%) and recruitment of an ERAS^®^ dedicated nurse (76%).

The above perceptions did not differ based on the respondent’s sex, years in practice, or case-mix. In contrast, surgeons with institutional care pathways (*p* = 0.043) and those with an annual PD volume <20 PDs/ year (*p* = 0.01) found difficulties in data collection and periodic audit. In addition, surgeons with an annual PD volume <20 PDs/year reported lack in administrative support (*p* < 0.001) and identified difficulties to collaborate with the members of the multidisciplinary team (*p* = 0.03) and employ an ERAS^®^ dedicated nurse (*p* = 0.001) as significant barriers to successful implementation and sustainability of peri-PD ERAS^®^ pathways. Surgeons from South America also reported higher lack of administrative support (*p* = 0.040) and reluctance amongst healthcare practitioners to change practice (*p* = 0.005) compared to other geographical regions (Supplementary Tables 5 A–F).

## Discussion

**Fig. 6:**
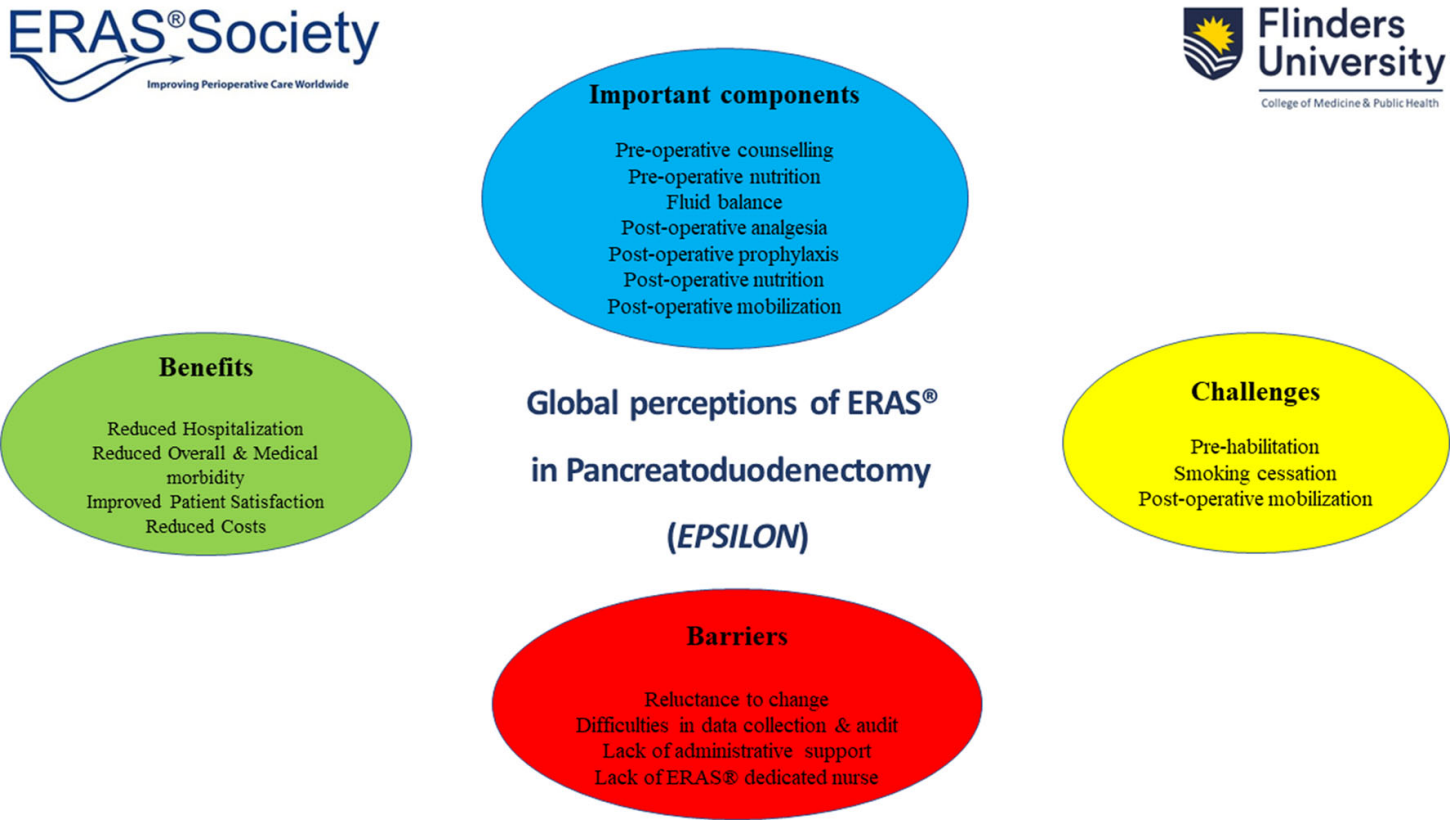
Graphical summary of the findings of EPSILON

PD remains the standard curative procedure for malignancies of the pancreatic head and peri-ampullary region [15]. Although refinements in the procedure, and peri-operative care practices, over the last few decades have resulted in a reduction in peri-operative mortality [16, 17], high post-operative morbidity continues to challenge pancreatic surgeons [18]. This international survey was carried out to obtain an understanding of the awareness, perceptions, practice of, and barriers to implementing protocols designed to enhance the recovery of patients undergoing PD around the world. The survey highlights the current low global utilisation of ERAS^®^ in PD with only 38% of respondents volunteering the use of post-PD pathways in their own practice. The findings of the study (Fig. 6) also demonstrate a shared appreciation of the benefits of compliance to ERAS^®^ in the peri-PD period amongst the respondents. Additionally, it also highlights variations in the perceptions of the importance of the individual components of peri-PD ERAS^®^ pathways and challenges to their application, as well as facilitators and barriers to the implementation and sustainability of peri-PD ERAS^®^ pathways based on the sex, geographical location, years in practice, case-mix, and annual PD volume.

Over the years, there have been numerous attempts to improve the peri-operative experience of the patients using enhanced recovery pathways [19, 20]. ERAS^®^ protocols were integrated into post-pancreatectomy care [4, 5] with an emphasis on creation of a supportive environment which not only ameliorates surgical stress, but also enables an optimal peri-operative experience for the patient [21]. In addition to reducing the post-operative length of stay [6, 7, 22–28], ERAS^®^ pathways have been shown to reduce overall morbidity [7, 23] major [28] and minor complications [6, 25, 27], DGE [6, 7, 25, 27], infectious complications [6, 26], without an increase in major complications, readmissions, reoperations or mortality [6, 7, 23–27]. However, despite evidence to the contrary, ERAS^®^ implementation and subsequent compliance remains far from optimal, especially in the field of pancreatic surgery [9].

The issue with PD has been the general tendency to proceed from diagnosis to surgery as quickly as possible, especially owing to the known influence of delays in surgery on outcomes of cancer (the most common indication for PD). Thus, we lack Level-1 evidence for pre-habilitation programs in PD. The growing utilisation of neoadjuvant therapy in patients undergoing PD for pancreatic cancer [29] will likely present an opportunity to introduce pre-habilitation in a meaningful way. The authors acknowledge that at the present time, a comprehensive pre-habilitation program requires financial investments [30]. However a value-based framework with bundled payments for the entire trajectory of care needs to be put in place, which would offset the initial costs of set-up [31]. Extrapolating the cost–benefit of such interventions from other surgical sub-specialties as well as the early experiences from centres that have already introduced them for pancreatic surgery can provide the global impetus to establish these units for pancreatic surgery.

Though believed to be multi-factorial, the reasons behind non-compliance can be broadly classified into provider (surgeon)-, and patient-, related. Surgeon reluctance stems from the fact that ERAS^®^ challenges many deeply-entrenched surgical dogmas [32]. In order to circumvent many of these hurdles, the ERAS^®^ society recommends the ERAS^®^ Implementation Process (EIP), a systematic training program which consists of four specific workshops over 8–10 months [33]. It has been previously reported that a multidisciplinary team needs a certain number of cases over a period of time to reach a satisfactory level of protocol adherence [34]. The ERAS^®^ Interactive Audit System (EIAS), an online interactive software, can be used to periodically assess compliance with the guidelines, and to objectively ascertain its impact on outcomes [33]. Interestingly, Roulin et al. [35] noted that non-compliance in the long-term is usually on account of patient-related factors / occurrence of complications, which is medically justified. Therefore, it is of paramount importance that local evidence-practice gaps are identified as a prequel to adapt evidence to the local circumstances so that measures can be undertaken to increase the provider/clinician involvement using periodic staff education sessions, incorporation of reminder systems, and audit and feedback [36].

An important message from this study is the identification of barriers to uniform implementation of peri-PD ERAS^®^ pathways, which include reluctance to change amongst healthcare practitioners, lack of multidisciplinary collaboration, data collection and audit, recruitment of an ERAS^®^ dedicated nurse and lack of administrative support. The former may be overcome by improving communication regarding the benefits of ERAS^®^ as well as focussed, periodic training and education sessions. Regarding the latter, qualitative studies exploring patient experiences from the use of ERAS^®^ as well as cost–benefit analyses, post-implementation, will help with building a business case to justify and encourage the investment needed to support the more widespread establishment of ERAS^®^ protocols in PD.

There are several limitations to this study which remain an inherent concern in self-reported surveys such as this [37]. Firstly, the survey relied on health care workers’ self-reports of their perceptions, experience and practice patterns. Secondly, expectedly, missing responses were occasionally encountered. The study is unable to establish a denominator in terms of the global practice of peri-PD ERAS^®^. However, the latter was not an objective of the survey. The overall lower response rate despite the survey being distributed via two of the most prominent societies whose membership is involved in the care of patients undergoing PD, namely, the ERAS^®^ society and the IHPBA, possibly reflects the low global utilisation of ERAS in PD— which was the one of the objectives of this survey, namely, to understand barriers to ERAS^®^ implementation. This survey was designed to identify perceptions, and not necessarily practice. Understanding perceptions is important to develop strategies aimed at education and training targeting practice-improving changes. Finally, there appears to be a clear variation in the geographical location of respondents favouring Europe. Whether this is a reflection of the true global practice of peri-PD ERAS^®^, or the membership of the societies through which the survey was disseminated cannot be conclusively ascertained.

In conclusion, the EPSILON survey highlighted global clinician perceptions regarding the benefits of compliance to peri-PD ERAS^®^, the importance of individual components [5] and challenges to their application. It not only helped identify perceived facilitators, but also challenges and barriers, to the implementation and sustainability of these pathways. The findings of this survey will help reinforce the benefit of enhancing recovery in patients undergoing PD by informing the surgical readership of the perceptions amongst the global fraternity. The survey will also empower the ERAS^®^ society in the provision of specific areas to focus on. The importance of multidisciplinary work is already the backbone of the implementation of ERAS^®^ through the EIP, but a specific module on multidisciplinary work with role-play could be added. Moreover, further efforts aimed at empowering patients must be encouraged with the active collection of PROMS (Patient-reported outcomes measures) and PREMS (Patient-reported experience measures) within the EIAS, which can serve to assess appropriateness of care, in terms of ‘what matters to patients’ [38]. PROM integration in clinical practice not only improves communication between patient and clinician, quality of care, patient experience, but has been associated with reduced *emergency department* visits and improved oncologic outcomes [38, 39]. The value of PROMs in pancreatic cancer has been previously reported by Maharaj et al. [40]. The development of these resources is essential to support clinicians and institutes who are keen to adopt ERAS^®^ in their routine pancreatic surgery practice guided by the ultimate desire to improve patient outcomes and experience.

### Supplementary Information

Below is the link to the electronic supplementary material.

Supplementary file 1 (DOCX 97 kb)

Supplementary file 2 (DOCX 40 kb)
