# Supplementary material for: Global Perceptions on ERAS® in Pancreatoduodenectomy
Source: World J Surg. 2023 Oct 3;47(12):2977–89. doi: 10.1007/s00268-023-07198-9 (PMC10694106; doi:10.1007/s00268-023-07198-9)
Supplement: Supplementary file 1 — (DOCX 97 kb) [file 268_2023_7198_MOESM1_ESM.docx]

**Supplementary Tables**

- **Perceptions on benefits of compliance to peri-PD ERAS®**

**Supplementary Table 1A:** **Perceptions on benefits of compliance to peri-PD ERAS® by sex (Abbreviations: ERAS – enhanced recovery after surgery; PD – pancreatoduodenectomy)**

| **Characteristic** | **N** | **Overall**, N = 140 | **Male**, N = 121 | **Female**, N = 19 | **p-value** |
| --- | --- | --- | --- | --- | --- |
| 1.Reduce length of stay | 137 | 8.0 (7.0, 9.0) | 8.0 (7.0, 9.0) | 8.0 (7.0, 8.0) | 0.5 |
| 2.Reduce overall complications | 138 | 8.0 (7.0, 10.0) | 8.0 (7.0, 10.0) | 8.0 (7.0, 10.0) | 0.6 |
| 3.Reduce pancreatic surgery-specific complications | 136 | 7.0 (5.0, 9.0) | 7.0 (5.0, 8.0) | 8.0 (7.0, 9.0) | 0.058 |
| 4.Reduce medical complications | 134 | 8.0 (7.0, 9.8) | 8.0 (7.0, 9.0) | 8.0 (7.0, 10.0) | 0.4 |
| 5.Reduce post-PD mortality | 137 | 7.0 (6.0, 9.0) | 7.0 (5.2, 9.0) | 7.0 (7.0, 9.5) | 0.5 |
| 6.Improve patient satisfaction | 134 | 8.0 (7.0, 9.0) | 8.0 (7.0, 9.0) | 8.0 (7.5, 9.5) | 0.4 |
| 7.Decrease costs | 137 | 8.0 (6.0, 9.0) | 7.5 (6.0, 9.0) | 8.0 (7.0, 8.5) | 0.7 |
| 8.Improve oncological outcomes | 133 | 7.0 (4.0, 8.0) | 7.0 (4.0, 8.0) | 8.0 (5.5, 9.0) | 0.3 |

*Data are presented as median and (25^th^-75^th^) percentiles. P values are calculated from* Mann-Whitney U test.

**Supplementary Table 1B: Perceptions on benefits of compliance to peri-PD ERAS® by geographical area (Abbreviations: ERAS – enhanced recovery after surgery; PD – pancreatoduodenectomy)**

| **Characteristic** | **N** | **Overall**, N = 140 | **Europe**, N = 63 | **Asia/Oceania**, N = 42 | **North America**, N = 24 | **North America**  N = 35 | **p-value** |
| --- | --- | --- | --- | --- | --- | --- | --- |
| 1.Reduce length of stay | 137 | 8.0 (7.0, 9.0) | 8.0 (7.0, 9.0) | 8.0 (7.0, 9.8) | 7.0 (7.0, 8.0) | 8.0 (7.0,10.0) | 0.3 |
| 2.Reduce overall complications | 138 | 8.0 (7.0, 10.0) | 8.0 (7.0, 9.8) | 8.0 (7.0, 9.8) | 8.0 (7.0, 10.0) | 8.0 (6.5, 9.5) | 0.9 |
| 3.Reduce pancreatic surgery-specific complications | 136 | 7.0 (5.0, 9.0) | 7.0 (5.0, 9.0) | 6.0 (4.0, 9.0) | 8.0 (5.0, 8.5) | 8.0 (6.2, 8.0) | 0.8 |
| 4.Reduce medical complications | 134 | 8.0 (7.0, 9.8) | 8.0 (7.0, 9.0) | 8.0 (7.0, 10.0) | 7.5 (6.0, 8.8) | 7.0 (6.5, 10.0) | 0.5 |
| 5.Reduce post-PD mortality | 137 | 7.0 (6.0, 9.0) | 7.0 (6.0, 9.0) | 8.0 (5.0, 9.0) | 7.0 (6.0, 9.0) | 8.0 (7.0, 8.5) | 0.9 |
| 6.Improve patient satisfaction | 134 | 8.0 (7.0, 9.0) | 8.0 (7.0, 9.0) | 8.0 (7.5, 10.0) | 7.0 (5.5, 8.0) | 8.0 (6.5, 10.0) | 0.078 |
| 7.Decrease costs | 137 | 8.0 (6.0, 9.0) | 8.0 (6.2, 9.0) | 8.0 (6.0, 9.0) | 7.0 (5.5, 8.0) | 7.0 (6.0, 9.0) | 0.7 |
| 8.Improve oncological outcomes | 133 | 7.0 (4.0, 8.0) | 7.0 (5.0, 8.0) | 6.0 (4.0, 8.0) | 6.0 (5.0, 8.5) | 6.0 (4.5, 7.5) | >0.9 |

*Data are presented as median and (25^th^-75^th^) percentiles. P values are calculated from* Kruskal-Wallis test.

**Supplementary Table 1C: Perceptions on benefits of compliance to peri-PD ERAS® by years in practice (Abbreviations: ERAS – enhanced recovery after surgery; PD – pancreatoduodenectomy)**

| **Characteristic** | **N** | **Overall**, N = 140 | **<10 Years**, N = 50 | **10-20 Years**, N = 54 | **>20 Years**, N = 36 | **p-value** |
| --- | --- | --- | --- | --- | --- | --- |
| 1.Reduce length of stay | 137 | 8.0 (7.0, 9.0) | 8.0 (7.0, 10.0) | 7.0 (6.0, 8.0) | 8.0 (7.0, 9.0) | 0.073 |
| 2.Reduce overall complications | 138 | 8.0 (7.0, 10.0) | 8.0 (7.0, 10.0) | 8.0 (7.0, 9.0) | 9.0 (7.0, 10.0) | 0.7 |
| 3.Reduce pancreatic surgery-specific complications | 136 | 7.0 (5.0, 9.0) | 6.0 (4.0, 8.0) | 8.0 (5.0, 8.0) | 8.0 (7.0, 9.0) | 0.079 |
| 4.Reduce medical complications | 134 | 8.0 (7.0, 9.8) | 7.0 (6.0, 9.0) | 8.0 (7.0, 9.0) | 8.0 (7.0, 10.0) | 0.061 |
| 5.Reduce post-PD mortality | 137 | 7.0 (6.0, 9.0) | 7.0 (5.0, 9.0) | 8.0 (5.2, 9.0) | 8.0 (6.5, 10.0) | 0.12 |
| 6.Improve patient satisfaction | 134 | 8.0 (7.0, 9.0) | 8.0 (6.0, 9.0) | 8.0 (7.0, 9.0) | 9.0 (7.5, 10.0) | 0.2 |
| 7.Decrease costs | 137 | 8.0 (6.0, 9.0) | 8.0 (7.0, 9.0) | 7.5 (6.0, 9.0) | 7.0 (6.0, 9.0) | 0.3 |
| 8.Improve oncological outcomes | 133 | 7.0 (4.0, 8.0) | 6.0 (4.0, 8.0) | 7.0 (4.0, 8.0) | 7.5 (5.2, 9.0) | 0.092 |

*Data are presented as median and (25^th^-75^th^) percentiles. P values are calculated from* Kruskal-Wallis test.

**Supplementary Table 1D: Perceptions on benefits of compliance to peri-PD ERAS® by case-mix (Abbreviations: ERAS – enhanced recovery after surgery; HPB – hepato-pancreato-biliary; PD – pancreatoduodenectomy)**

| **Characteristic** | **N** | **HPB Surgery**, N = 96 | **General Surgery**, N = 25 | **Pancreatic Surgery**, N = 11 | **Others**, N = 8 | **p-value** |
| --- | --- | --- | --- | --- | --- | --- |
| 1.Reduce length of stay | 137 | 8.0 (7.0, 10.0) | 8.0 (7.0, 8.0) | 8.0 (7.0, 10.0) | 6.5 (5.0, 7.2) | 0.066 |
| 2.Reduce overall complications | 138 | 8.0 (7.0, 10.0) | 8.0 (7.0, 9.0) | 8.0 (7.0, 10.0) | 7.5 (5.8, 8.0) | 0.6 |
| 3.Reduce pancreatic surgery-specific complications | 136 | 8.0 (5.0, 9.0) | 6.0 (5.0, 8.0) | 7.0 (5.0, 9.5) | 5.0 (4.0, 7.2) | 0.4 |
| 4.Reduce medical complications | 134 | 8.0 (6.8, 9.2) | 8.0 (7.0, 9.0) | 9.5 (8.2, 10.0) | 8.0 (7.0, 8.0) | 0.3 |
| 5.Reduce post-PD mortality | 137 | 8.0 (6.0, 10.0) | 7.0 (5.0, 8.2) | 8.0 (7.0, 9.5) | 6.0 (5.0, 8.2) | 0.4 |
| 6.Improve patient satisfaction | 134 | 8.0 (7.0, 9.0) | 8.0 (7.0, 9.0) | 8.5 (7.0, 10.0) | 7.0 (5.0, 8.0) | 0.11 |
| 7.Decrease costs | 137 | 8.0 (6.0, 9.0) | 7.5 (6.0, 9.0) | 8.0 (7.0, 8.5) | 5.5 (5.0, 7.2) | 0.2 |
| 8.Improve oncological outcomes | 133 | 7.0 (5.0, 8.0) | 6.5 (4.0, 8.0) | 6.5 (3.8, 9.8) | 5.5 (4.0, 7.0) | 0.5 |

*Data are presented as median and (25^th^-75^th^) percentiles. P values are calculated from* Kruskal-Wallis test.

**Supplementary Table 1E: Perceptions on benefits of compliance to peri-PD ERAS® by annual PD volume (Abbreviations: ERAS – enhanced recovery after surgery; PD – pancreatoduodenectomy)**

| **Characteristic** | **N** | **<20**, N = 33 | **20-50**, N = 57 | **>50**, N = 50 | **p-value** |
| --- | --- | --- | --- | --- | --- |
| 1.Reduce length of stay | 137 | 8.0 (7.0, 9.0) | 8.0 (7.0, 9.0) | 8.0 (7.0, 9.0) | >0.9 |
| 2.Reduce overall complications | 138 | 8.0 (7.0, 9.0) | 8.0 (7.8, 10.0) | 8.0 (7.0, 10.0) | 0.4 |
| 3.Reduce pancreatic surgery-specific complications | 136 | 8.0 (5.8, 8.2) | 7.0 (5.0, 9.0) | 7.0 (4.0, 8.0) | 0.8 |
| 4.Reduce medical complications | 134 | 8.0 (6.0, 9.0) | 8.0 (7.0, 9.5) | 8.0 (7.0, 10.0) | 0.8 |
| 5.Reduce post-PD mortality | 137 | 7.5 (6.0, 9.0) | 7.5 (6.0, 9.2) | 7.0 (5.0, 9.0) | 0.7 |
| 6.Improve patient satisfaction | 134 | 8.0 (6.5, 9.5) | 8.0 (7.0, 9.0) | 8.0 (6.5, 9.0) | 0.5 |
| 7.Decrease costs | 137 | 8.0 (6.8, 9.0) | 7.0 (5.0, 9.0) | 8.0 (7.0, 9.0) | 0.3 |
| 8.Improve oncological outcomes | 133 | 7.0 (4.0, 8.0) | 7.0 (5.0, 9.0) | 5.5 (4.0, 8.0) | 0.3 |

*Data are presented as median and (25^th^-75^th^) percentiles. P values are calculated from* Kruskal-Wallis test.

**Supplementary Table 2F: Perceptions on benefits of compliance to peri-PD ERAS® by unit’s PD patient care routine care practice (Abbreviations: ERAS – enhanced recovery after surgery; CP – clinical pathways; PD – pancreatoduodenectomy)**

| **Characteristic** | **N** | **ERAS® Pathways**, N = 45 | **Post-PD CP to Enhance Recovery**, N = 53 | **Management at Surgeon’s Discretion**, N = 42 | **p-value** |
| --- | --- | --- | --- | --- | --- |
| 1.Reduce length of stay | 137 | 8.0 (7.0, 9.0) | 8.0 (7.0, 9.0) | 8.0 (7.0, 9.2) | 0.6 |
| 2.Reduce overall complications | 138 | 9.0 (7.8, 10.0) | 8.0 (7.0, 10.0) | 8.0 (7.0, 9.0) | 0.2 |
| 3.Reduce pancreatic surgery-specific complications | 136 | 8.0 (5.0, 9.0) | 7.0 (5.0, 9.0) | 7.0 (5.0, 8.0) | 0.4 |
| 4.Reduce medical complications | 134 | 8.0 (7.0, 10.0) | 8.0 (7.0, 9.0) | 8.0 (6.8, 9.0) | 0.6 |
| 5.Reduce post-PD mortality | 137 | 8.0 (6.0, 10.0) | 7.0 (5.0, 9.0) | 8.0 (6.0, 9.0) | 0.4 |
| 6.Improve patient satisfaction | 134 | 9.0 (8.0, 9.0) | 8.0 (6.2, 9.0) | 8.0 (6.0, 9.0) | 0.089 |
| 7.Decrease costs | 137 | 8.0 (7.0, 9.0) | 8.0 (5.0, 9.0) | 7.0 (6.0, 9.0) | 0.4 |
| 8.Improve oncological outcomes | 133 | 7.0 (5.0, 8.0) | 6.0 (4.0, 8.0) | 7.0 (5.0, 8.0) | 0.7 |

*Data are presented as median and (25^th^-75^th^) percentiles. P values are calculated* from Kruskal-Wallis test.

**Perceived importance of individual ERAS components**

**Supplementary Table 2A: Perceptions on importance of individual components of peri-PD ERAS® by sex (Abbreviations: ERAS – enhanced recovery after surgery; PD – pancreatoduodenectomy)**

| **Characteristic** | **N** | **Overall**, N = 140 | **Male**, N = 121 | **Female**, N = 19 | **p-value** |
| --- | --- | --- | --- | --- | --- |
| 1.Preoperative counselling | 136 | 9.0 (7.0, 10.0) | 8.0 (7.0, 10.0) | 9.0 (8.0, 10.0) | 0.2 |
| 2.Pre-habilitation | 135 | 8.0 (7.0, 9.0) | 8.0 (7.0, 9.0) | 8.5 (8.0, 9.0) | 0.4 |
| 3.Routine PBD | 136 | 5.0 (2.0, 6.0) | 5.0 (2.0, 6.0) | 5.0 (3.0, 6.8) | 0.8 |
| 4.Smoking cessation | 135 | 8.0 (6.0, 9.0) | 8.0 (6.0, 9.0) | 8.0 (7.0, 9.0) | 0.6 |
| 5.Pre-op nutritional intervention | 136 | 9.0 (7.0, 9.0) | 8.0 (7.0, 9.0) | 9.0 (8.2, 10.0) | 0.039 |
| 6.Carbohydrate loading | 136 | 7.0 (6.0, 8.0) | 7.0 (5.0, 8.0) | 8.0 (6.2, 8.0) | 0.2 |
| 7.Pre-anaesthetic medication | 134 | 7.0 (5.0, 8.8) | 7.0 (5.8, 9.0) | 6.5 (4.0, 8.0) | 0.3 |
| 8.Thrombotic prophylaxis | 134 | 8.0 (7.0, 9.0) | 8.0 (7.0, 9.0) | 8.5 (6.2, 9.0) | 0.9 |
| 9.Antimicrobial prophylaxis | 135 | 8.0 (7.0, 9.0) | 8.0 (7.0, 9.0) | 9.0 (8.0, 9.8) | 0.4 |
| 10.Analgesia | 134 | 9.0 (8.0, 10.0) | 9.0 (8.0, 10.0) | 9.0 (9.0, 10.0) | 0.2 |
| 11.PONV prophylaxis | 135 | 9.0 (8.0, 9.0) | 8.0 (8.0, 9.0) | 9.0 (8.0, 9.8) | 0.3 |
| 12.Avoiding hypothermia | 134 | 8.0 (7.0, 9.0) | 8.0 (7.0, 9.0) | 9.0 (8.0, 10.0) | 0.11 |
| 13.Postoperative glycaemic control | 135 | 8.0 (7.0, 9.0) | 8.0 (7.0, 9.0) | 9.0 (8.0, 9.8) | 0.12 |
| 14.Nasogastric (NG) intubation | 134 | 7.0 (5.0, 9.0) | 7.0 (5.0, 9.0) | 8.0 (5.2, 9.0) | 0.3 |
| 15.Fluid balance | 132 | 9.0 (8.0, 10.0) | 9.0 (8.0, 10.0) | 9.0 (8.2, 10.0) | 0.2 |
| 16.Peri-anastomotic drainage | 132 | 7.5 (5.0, 9.0) | 7.5 (5.0, 9.0) | 7.5 (6.0, 9.0) | 0.8 |
| 17.Use of somatostatin analogues | 135 | 4.0 (0.0, 6.0) | 4.0 (0.0, 6.0) | 6.0 (0.8, 7.0) | 0.3 |
| 18.Urinary drainage | 132 | 7.0 (5.0, 8.0) | 6.0 (5.0, 8.0) | 8.0 (6.0, 9.0) | 0.048 |
| 19.Stimulation of bowel movement | 131 | 7.0 (5.0, 8.5) | 7.0 (5.0, 8.0) | 7.5 (6.2, 9.0) | 0.14 |
| 20.Post-operative nutrition | 134 | 9.0 (7.0, 10.0) | 9.0 (7.0, 9.2) | 8.5 (8.0, 10.0) | 0.7 |
| 21.Post-operative mobilization | 133 | 9.0 (8.0, 10.0) | 9.0 (8.0, 10.0) | 9.0 (8.2, 10.0) | >0.9 |
| 22.Minimally invasive PD | 132 | 6.0 (3.0, 8.0) | 5.0 (3.0, 8.0) | 6.0 (5.0, 8.0) | 0.2 |

*Data are presented as median and (25^th^-75^th^) percentiles. P values are calculated from* Mann-Whitney U test.

**Supplementary Table 2B: Perceptions on importance of individual components of peri-PD ERAS® by geographical area (Abbreviations: ERAS – enhanced recovery after surgery; PD – pancreatoduodenectomy)**

| **Characteristic** | **N** | **Overall**, N = 140 | **Europe**, N = 63 | **Asia/Oceania**, N = 42 | **North America**,  N = 24 | **South America**,  N = 11 | **p-value** |
| --- | --- | --- | --- | --- | --- | --- | --- |
| 1.Preoperative counselling | 136 | 9.0 (7.0, 10.0) | 9.0 (8.0, 9.0) | 9.0 (8.0, 10.0) | 8.0 (7.0, 9.0) | 10.0 (8.0, 10.0) | 0.11 |
| 2.Pre-habilitation | 135 | 8.0 (7.0, 9.0) | 8.0 (7.0, 9.0) | 8.5 (7.8, 10.0) | 8.0 (6.0, 9.0) | 9.0 (8.5, 10.0) | 0.063 |
| 3.Routine PBD | 136 | 5.0 (2.0, 6.0) | 5.0 (3.0, 7.0) | 5.0 (0.0, 5.2) | 5.0 (2.0, 7.0) | 6.0 (4.0, 6.0) | 0.4 |
| 4.Smoking cessation | 135 | 8.0 (6.0, 9.0) | 7.0 (5.0, 8.0) | 9.0 (7.0, 10.0) | 7.0 (6.0, 8.5) | 9.0 (7.5,10.0) | <0.001 |
| 5.Pre-op nutritional intervention | 136 | 9.0 (7.0, 9.0) | 9.0 (7.2, 9.0) | 8.0 (7.0, 10.0) | 8.0 (6.5, 9.0) | 10.0 (8.5, 10.0) | 0.15 |
| 6.Carbohydrate loading | 136 | 7.0 (6.0, 8.0) | 7.0 (5.0, 8.0) | 8.0 (6.0, 8.0) | 7.0 (5.0, 7.5) | 7.0 (6.5, 8.0) | 0.3 |
| 7.Pre-anaesthetic medication | 134 | 7.0 (5.0, 8.8) | 7.0 (6.0, 9.0) | 7.0 (5.8, 9.0) | 7.0 (5.0, 8.0) | 6.5 (2.8, 8.0) | 0.7 |
| 8.Thrombotic prophylaxis | 134 | 8.0 (7.0, 9.0) | 8.0 (7.0, 9.0) | 8.0 (7.0, 10.0) | 8.0 (7.0, 9.0) | 9.0 (7.0, 10.0) | 0.7 |
| 9.Antimicrobial prophylaxis | 135 | 8.0 (7.0, 9.0) | 8.0 (7.0, 9.0) | 9.0 (7.0, 10.0) | 8.0 (7.5, 9.0) | 8.0 (8.0, 9.5) | 0.4 |
| 10.Analgesia | 134 | 9.0 (8.0, 10.0) | 9.0 (8.0, 9.0) | 9.0 (8.0, 10.0) | 8.0 (8.0, 9.5) | 10.0 (9.5, 10.0) | 0.007 |
| 11.PONV prophylaxis | 135 | 9.0 (8.0, 9.0) | 8.0 (8.0, 9.0) | 9.0 (7.0, 10.0) | 8.0 (7.0, 9.0) | 10.0 (8.5, 10.0) | 0.2 |
| 12.Avoiding hypothermia | 134 | 8.0 (7.0, 9.0) | 8.0 (7.0, 9.0) | 9.0 (8.0, 10.0) | 8.0 (7.0, 8.5) | 10.0 (7.5, 10.0) | 0.052 |
| 13.Postoperative glycaemic control | 135 | 8.0 (7.0, 9.0) | 8.0 (7.0, 9.0) | 8.5 (8.0, 9.0) | 8.0 (7.0, 8.5) | 9.0 (7.5, 10.0) | 0.2 |
| 14.Nasogastric (NG) intubation | 134 | 7.0 (5.0, 9.0) | 8.0 (6.0, 9.0) | 6.5 (4.8, 9.2) | 7.0 (3.5, 8.5) | 7.0 (5.0, 8.5) | 0.8 |
| 15.Fluid balance | 132 | 9.0 (8.0, 10.0) | 9.0 (8.0, 10.0) | 9.0 (8.0, 10.0) | 8.0 (7.0, 9.0) | 8.0 (7.0, 9.8) | 0.3 |
| 16.Peri-anastomotic drainage | 132 | 7.5 (5.0, 9.0) | 7.0 (6.0, 9.0) | 8.0 (4.0, 9.0) | 8.0 (6.0, 8.5) | 7.0 (5.5, 8.5) | >0.9 |
| 17.Use of somatostatin analogues | 135 | 4.0 (0.0, 6.0) | 5.0 (2.0, 7.0) | 3.5 (0.0, 5.2) | 3.0 (2.0, 5.0) | 4.0 (2.0, 6.0) | 0.3 |
| 18.Urinary drainage | 132 | 7.0 (5.0, 8.0) | 7.0 (5.0, 8.0) | 7.0 (4.8, 8.0) | 6.0 (3.2, 7.0) | 7.0 (5.0, 8.0) | 0.6 |
| 19.Stimulation of bowel movement | 131 | 7.0 (5.0, 8.5) | 7.0 (5.0, 9.0) | 7.0 (5.0, 8.0) | 6.0 (4.0, 7.0) | 8.0 (7.0, 9.5) | 0.082 |
| 20.Post-operative nutrition | 134 | 9.0 (7.0, 10.0) | 9.0 (7.8, 9.0) | 9.0 (7.8, 10.0) | 8.0 (6.5, 9.0) | 10.0 (9.0, 10.0) | 0.009 |
| 21.Post-operative mobilization | 133 | 9.0 (8.0, 10.0) | 9.0 (9.0, 10.0) | 9.0 (8.8, 10.0) | 9.0 (8.0, 10.0) | 10.0 (9.0, 10.0) | 0.4 |
| 22.Minimally invasive PD | 132 | 6.0 (3.0, 8.0) | 6.0 (4.5, 8.0) | 6.0 (2.0, 7.0) | 5.0 (2.5, 7.0) | 6.0 (3.5-8.0) | 0.7 |

*Data are presented as median and (25^th^-75^th^) percentiles. P values are calculated* from Kruskal-Wallis test.

**Supplementary Table 2C: Perceptions on importance of individual components of peri-PD ERAS® by years in practice (Abbreviations: ERAS – enhanced recovery after surgery; PD – pancreatoduodenectomy)**

| **Characteristic** | **N** | **Overall**, N = 140 | **<10 Years**, N = 50 | **10-20 Years**, N = 54 | **>20 Years**, N = 36 | **p-value** |
| --- | --- | --- | --- | --- | --- | --- |
| 1.Preoperative counselling | 136 | 9.0 (7.0, 10.0) | 8.0 (7.0, 10.0) | 9.0 (7.0, 10.0) | 9.0 (8.0, 10.0) | 0.7 |
| 2.Pre-habilitation | 135 | 8.0 (7.0, 9.0) | 8.0 (7.0, 10.0) | 8.0 (6.0, 9.0) | 9.0 (7.0, 10.0) | 0.2 |
| 3.Routine PBD | 136 | 5.0 (2.0, 6.0) | 5.0 (0.0, 7.0) | 5.0 (2.0, 6.0) | 5.0 (2.0, 6.5) | >0.9 |
| 4.Smoking cessation | 135 | 8.0 (6.0, 9.0) | 7.0 (6.0, 9.0) | 8.0 (6.0, 9.0) | 8.5 (7.0, 9.0) | 0.036 |
| 5.Pre-op nutritional intervention | 136 | 9.0 (7.0, 9.0) | 8.0 (7.0, 10.0) | 8.0 (7.0, 9.0) | 9.0 (8.0, 10.0) | 0.3 |
| 6.Carbohydrate loading | 136 | 7.0 (6.0, 8.0) | 7.0 (6.0, 8.0) | 7.0 (5.0, 8.0) | 7.0 (6.0, 9.0) | 0.4 |
| 7.Pre-anaesthetic medication | 134 | 7.0 (5.0, 8.8) | 7.0 (5.0, 8.0) | 7.0 (5.0, 8.0) | 7.0 (7.0, 9.0) | 0.12 |
| 8.Thrombotic prophylaxis | 134 | 8.0 (7.0, 9.0) | 8.0 (7.0, 9.0) | 7.0 (6.0, 9.0) | 9.0 (8.0, 10.0) | 0.005 |
| 9.Antimicrobial prophylaxis | 135 | 8.0 (7.0, 9.0) | 8.0 (7.0, 9.0) | 8.0 (7.0, 9.0) | 9.0 (8.0, 10.0) | 0.054 |
| 10.Analgesia | 134 | 9.0 (8.0, 10.0) | 9.0 (8.0, 9.0) | 9.0 (8.0, 10.0) | 9.0 (8.0, 10.0) | 0.5 |
| 11.PONV prophylaxis | 135 | 9.0 (8.0, 9.0) | 8.0 (7.0, 9.0) | 8.0 (7.0, 9.0) | 9.0 (8.0, 10.0) | 0.042 |
| 12.Avoiding hypothermia | 134 | 8.0 (7.0, 9.0) | 8.0 (7.0, 9.0) | 8.0 (7.0, 9.0) | 9.0 (7.0, 10.0) | 0.13 |
| 13.Postoperative glycaemic control | 135 | 8.0 (7.0, 9.0) | 8.0 (7.0, 9.0) | 8.0 (7.0, 9.0) | 9.0 (8.0, 10.0) | 0.13 |
| 14.Nasogastric (NG) intubation | 134 | 7.0 (5.0, 9.0) | 7.0 (6.0, 9.0) | 7.0 (4.0, 9.0) | 7.0 (5.0, 8.0) | 0.5 |
| 15.Fluid balance | 132 | 9.0 (8.0, 10.0) | 9.0 (8.0, 10.0) | 9.0 (8.0, 9.2) | 9.0 (8.0, 10.0) | 0.9 |
| 16.Peri-anastomotic drainage | 132 | 7.5 (5.0, 9.0) | 8.0 (5.5, 9.0) | 7.0 (4.8, 9.0) | 8.0 (7.0, 9.0) | 0.3 |
| 17.Use of somatostatin analogues | 135 | 4.0 (0.0, 6.0) | 3.0 (0.0, 6.0) | 5.0 (2.0, 6.0) | 5.0 (0.0, 7.5) | 0.2 |
| 18.Urinary drainage | 132 | 7.0 (5.0, 8.0) | 7.0 (4.5, 8.0) | 6.0 (3.0, 8.0) | 7.0 (5.0, 8.2) | 0.081 |
| 19.Stimulation of bowel movement | 131 | 7.0 (5.0, 8.5) | 6.0 (3.2, 8.0) | 7.0 (6.0, 8.0) | 8.0 (5.0, 9.0) | 0.067 |
| 20.Post-operative nutrition | 134 | 9.0 (7.0, 10.0) | 8.0 (8.0, 10.0) | 9.0 (7.0, 9.0) | 9.0 (7.0, 10.0) | 0.6 |
| 21.Post-operative mobilization | 133 | 9.0 (8.0, 10.0) | 9.0 (8.0, 10.0) | 9.0 (8.0, 10.0) | 10.0 (9.0, 10.0) | 0.049 |
| 22.Minimally invasive PD | 132 | 6.0 (3.0, 8.0) | 6.0 (4.2, 8.0) | 5.0 (3.0, 8.0) | 6.0 (4.0, 7.0) | 0.3 |

*Data are presented as median and (25^th^-75^th^) percentiles. P values are calculated* from Kruskal-Wallis test.

**Supplementary Table 2D: Perceptions on importance of individual components of peri-PD ERAS® by case-mix (Abbreviations: ERAS – enhanced recovery after surgery; HPB – hepato-pancreato-biliary; PD – pancreatoduodenectomy)**

| **Characteristic** | **N** | **Overall**,  N = 140 | **HPB Surgery**,  N = 96 | **General Surgery**,  N = 25 | **Pancreatic Surgery**,  N = 11 | **Others**,  N = 8 | **p-value** |
| --- | --- | --- | --- | --- | --- | --- | --- |
| 1.Preoperative counselling | 136 | 9 (7.0, 10) | 9 (8.0, 10) | 8 (6.0, 9) | 8 (8.0, 9) | 8 (7.0, 10) | 0.093 |
| 2.Pre-habilitation | 135 | 8 (7.0, 9) | 8 (7.0, 10) | 8 (6.8, 9) | 8 (8.0, 9) | 8 (6.8, 8) | 0.8 |
| 3.Routine PBD | 136 | 5 (2.0, 6) | 5 (0.0, 6) | 5 (2.8, 6) | 4 (3.0, 8) | 6 (4.8, 6) | 0.6 |
| 4.Smoking cessation | 135 | 8 (6.0, 9) | 8 (6.0, 9) | 7 (6.0, 8) | 8 (6.2, 8) | 8 (7.2, 9) | 0.14 |
| 5.Pre-op nutritional intervention | 136 | 9 (7.0, 9) | 8 (7.0, 9) | 9 (8.0, 10) | 9 (8.5, 9) | 8 (8.0, 9) | 0.6 |
| 6.Carbohydrate loading | 136 | 7 (6.0, 8) | 7 (6.0, 8) | 8 (6.8, 8) | 7 (5.5, 8) | 6 (5.2, 7) | 0.6 |
| 7.Pre-anaesthetic medication | 134 | 7 (5.0, 9) | 7 (5.0, 9) | 7 (5.0, 8) | 7 (7.0, 9) | 6 (4.8, 8) | 0.6 |
| 8.Thrombotic prophylaxis | 134 | 8 (7.0, 9) | 8 (7.0, 10) | 8 (7.0, 9) | 9 (8.2, 9) | 7 (5.8, 9) | 0.4 |
| 9.Antimicrobial prophylaxis | 135 | 8 (7.0, 9) | 8 (7.0, 10) | 8 (7.0, 9) | 9 (7.5, 10) | 8 (7.0, 9) | 0.8 |
| 10.Analgesia | 134 | 9 (8.0, 10) | 9 (8.0, 10) | 9 (8.0, 10) | 8 (8.0, 9) | 8 (8.0, 9) | 0.7 |
| 11.PONV prophylaxis | 135 | 9 (8.0, 9) | 9 (8.0, 10) | 9 (7.8, 9) | 8 (8.0, 9) | 8 (5.2, 8) | 0.13 |
| 12.Avoiding hypothermia | 134 | 8 (7.0, 9) | 8 (7.0, 9) | 8 (8.0, 9) | 8 (7.0, 9) | 8 (8.0, 9) | 0.8 |
| 13.Postoperative glycaemic control | 135 | 8 (7.0, 9) | 8 (7.0, 9) | 8 (8.0, 9) | 8 (8.0, 9) | 8 (7.2, 9) | >0.9 |
| 14.Nasogastric (NG) intubation | 134 | 7 (5.0, 9) | 7 (5.0, 9) | 8 (5.0, 9) | 6 (5.2, 8) | 2 (2.0, 6) | 0.14 |
| 15.Fluid balance | 132 | 9 (8.0, 10) | 9 (8.0, 10) | 9 (8.0, 10) | 9 (8.2, 10) | 9 (8.0, 10) | 0.7 |
| 16.Peri-anastomotic drainage | 132 | 8 (5.0, 9) | 7 (5.0, 9) | 8 (7.0, 8) | 8 (7.0, 9) | 4 (3.0, 7) | 0.2 |
| 17.Use of somatostatin analogues | 135 | 4 (0.0, 6) | 4 (0.0, 6) | 5 (3.0, 6) | 5 (0.0, 7) | 4 (1.5, 5) | >0.9 |
| 18.Urinary drainage | 132 | 7 (5.0, 8) | 6 (5.0, 8) | 7 (4.0, 8) | 7 (7.0, 8) | 8 (5.2, 9) | 0.5 |
| 19.Stimulation of bowel movement | 131 | 7 (5.0, 8) | 7 (5.0, 9) | 7 (6.0, 8) | 7 (5.0, 8) | 6 (2.8, 8) | 0.8 |
| 20.Post-operative nutrition | 134 | 9 (7.0, 10) | 9 (8.0, 10) | 9 (8.0, 10) | 8 (6.2, 9) | 8 (7.0, 9) | 0.4 |
| 21.Post-operative mobilization | 133 | 9 (8.0, 10) | 9 (9.0, 10) | 9 (9.0, 10) | 9 (8.0, 9) | 9 (8.0, 10) | 0.5 |
| 22.Minimally invasive PD | 132 | 6 (3.0, 8) | 6 (3.0, 8) | 7 (5.2, 9) | 5 (4.0, 8) | 2 (1.5, 5) | 0.007 |

*Data are presented as median and (25^th^-75^th^) percentiles. P values are calculated* from Kruskal-Wallis test.

**Supplementary Table 2E: Perceptions on importance of individual components of peri-PD ERAS® by annual PD volume (Abbreviations: ERAS – enhanced recovery after surgery; PD – pancreatoduodenectomy)**

| **Characteristic** | **N** | **Overall**, N = 140 | **<20**, N = 33 | **20-50**, N = 57 | **>50**, N = 50 | **p-value** |
| --- | --- | --- | --- | --- | --- | --- |
| 1.Preoperative counselling | 136 | 9.0 (7.0, 10.0) | 9.0 (7.0, 10.0) | 8.5 (8.0, 10.0) | 9.0 (7.0, 9.0) | 0.9 |
| 2.Pre-habilitation | 135 | 8.0 (7.0, 9.0) | 8.5 (8.0, 10.0) | 8.0 (6.0, 9.8) | 8.0 (7.0, 9.0) | 0.2 |
| 3.Routine PBD | 136 | 5.0 (2.0, 6.0) | 5.0 (2.8, 6.0) | 5.0 (2.0, 6.0) | 5.0 (0.0, 7.0) | 0.6 |
| 4.Smoking cessation | 135 | 8.0 (6.0, 9.0) | 9.0 (7.8, 9.0) | 7.0 (6.0, 9.0) | 8.0 (6.0, 8.0) | 0.023 |
| 5.Pre-op nutritional intervention | 136 | 9.0 (7.0, 9.0) | 9.0 (8.0, 10.0) | 8.0 (7.0, 9.0) | 8.0 (6.0, 9.0) | 0.007 |
| 6.Carbohydrate loading | 136 | 7.0 (6.0, 8.0) | 7.0 (7.0, 9.0) | 7.0 (6.0, 8.0) | 7.0 (5.0, 8.0) | 0.15 |
| 7.Pre-anaesthetic medication | 134 | 7.0 (5.0, 8.8) | 8.0 (5.5, 9.0) | 7.0 (6.0, 9.0) | 7.0 (5.0, 8.0) | 0.7 |
| 8.Thrombotic prophylaxis | 134 | 8.0 (7.0, 9.0) | 9.0 (7.0, 10.0) | 8.0 (7.0, 9.0) | 8.0 (7.0, 9.0) | 0.5 |
| 9.Antimicrobial prophylaxis | 135 | 8.0 (7.0, 9.0) | 9.0 (8.0, 10.0) | 8.0 (7.0, 9.5) | 8.0 (7.0, 9.0) | 0.064 |
| 10.Analgesia | 134 | 9.0 (8.0, 10.0) | 9.5 (9.0, 10.0) | 9.0 (8.0, 10.0) | 8.0 (8.0, 9.0) | 0.005 |
| 11.PONV prophylaxis | 135 | 9.0 (8.0, 9.0) | 9.0 (8.0, 10.0) | 8.0 (7.2, 9.0) | 8.0 (8.0, 9.0) | 0.3 |
| 12.Avoiding hypothermia | 134 | 8.0 (7.0, 9.0) | 9.0 (8.0, 10.0) | 8.0 (7.0, 9.0) | 8.0 (7.0, 9.0) | 0.005 |
| 13.Postoperative glycaemic control | 135 | 8.0 (7.0, 9.0) | 9.0 (8.8, 10.0) | 8.0 (7.0, 9.0) | 8.0 (7.0, 9.0) | 0.001 |
| 14.Nasogastric (NG) intubation | 134 | 7.0 (5.0, 9.0) | 6.5 (5.0, 9.0) | 7.0 (6.0, 9.0) | 7.0 (5.0, 8.0) | 0.8 |
| 15.Fluid balance | 132 | 9.0 (8.0, 10.0) | 9.0 (8.0, 10.0) | 9.0 (8.0, 10.0) | 9.0 (8.0, 10.0) | 0.7 |
| 16.Peri-anastomotic drainage | 132 | 7.5 (5.0, 9.0) | 8.0 (6.0, 9.0) | 7.0 (4.8, 9.0) | 7.0 (5.8, 8.0) | 0.3 |
| 17.Use of somatostatin analogues | 135 | 4.0 (0.0, 6.0) | 5.0 (2.8, 6.2) | 4.0 (0.0, 6.0) | 4.5 (0.0, 6.2) | 0.4 |
| 18.Urinary drainage | 132 | 7.0 (5.0, 8.0) | 7.5 (5.8, 9.0) | 6.0 (3.0, 8.0) | 7.0 (5.0, 8.0) | 0.054 |
| 19.Stimulation of bowel movement | 131 | 7.0 (5.0, 8.5) | 8.0 (5.8, 9.2) | 7.0 (5.0, 9.0) | 6.0 (4.5, 7.5) | 0.030 |
| 20.Post-operative nutrition | 134 | 9.0 (7.0, 10.0) | 9.0 (8.0, 10.0) | 9.0 (8.0, 9.8) | 8.0 (7.0, 9.0) | 0.069 |
| 21.Post-operative mobilization | 133 | 9.0 (8.0, 10.0) | 9.0 (9.0, 10.0) | 9.0 (9.0, 10.0) | 9.0 (8.0, 9.0) | 0.015 |
| 22.Minimally invasive PD | 132 | 6.0 (3.0, 8.0) | 7.0 (5.0, 9.0) | 5.0 (3.0, 7.0) | 5.0 (2.0, 8.0) | 0.10 |

*Data are presented as median and (25^th^-75^th^) percentiles. P values are calculated* from Kruskal-Wallis test.

**Supplementary Table 2F: Perceptions on importance of individual components of peri-PD ERAS® by unit’s PD patient care routine care practice**

| **Characteristic** | **N** | **Overall**, N = 140 | **ERAS® Pathways**, N = 45 | **Post-PD CP to Enhance Recovery**, N = 53 | **Management at Surgeon’s Discretion**, N = 42 | **p-value** |
| --- | --- | --- | --- | --- | --- | --- |
| 1.Preoperative counselling | 136 | 9.0 (7.0, 10.0) | 9.0 (7.5, 10.0) | 8.0 (7.0, 10.0) | 8.5 (8.0, 10.0) | 0.8 |
| 2.Pre-habilitation | 135 | 8.0 (7.0, 9.0) | 8.0 (6.8, 9.0) | 8.0 (7.0, 9.5) | 8.5 (7.8, 10.0) | 0.4 |
| 3.Routine PBD | 136 | 5.0 (2.0, 6.0) | 5.0 (3.8, 7.2) | 4.0 (1.5, 6.0) | 5.0 (0.0, 6.2) | 0.2 |
| 4.Smoking cessation | 135 | 8.0 (6.0, 9.0) | 7.0 (6.0, 9.0) | 8.0 (5.5, 9.0) | 8.0 (7.0, 9.0) | 0.3 |
| 5.Pre-op nutritional intervention | 136 | 9.0 (7.0, 9.0) | 9.0 (7.0, 9.2) | 8.5 (7.8, 9.0) | 8.0 (7.0, 9.2) | >0.9 |
| 6.Carbohydrate loading | 136 | 7.0 (6.0, 8.0) | 7.0 (6.0, 9.0) | 7.0 (5.0, 8.0) | 7.0 (6.0, 8.0) | 0.9 |
| 7.Pre-anaesthetic medication | 134 | 7.0 (5.0, 8.8) | 7.0 (5.5, 9.0) | 7.0 (5.0, 8.0) | 7.0 (5.0, 8.0) | >0.9 |
| 8.Thrombotic prophylaxis | 134 | 8.0 (7.0, 9.0) | 8.0 (7.0, 9.0) | 9.0 (7.0, 9.0) | 8.0 (7.0, 9.0) | 0.7 |
| 9.Antimicrobial prophylaxis | 135 | 8.0 (7.0, 9.0) | 8.0 (7.0, 9.0) | 8.5 (7.0, 10.0) | 9.0 (7.5, 9.0) | 0.4 |
| 10.Analgesia | 134 | 9.0 (8.0, 10.0) | 9.0 (8.0, 10.0) | 9.0 (8.0, 10.0) | 9.0 (8.0, 10.0) | 0.3 |
| 11.PONV prophylaxis | 135 | 9.0 (8.0, 9.0) | 8.0 (8.0, 9.5) | 8.0 (7.0, 9.0) | 9.0 (8.0, 10.0) | 0.2 |
| 12.Avoiding hypothermia | 134 | 8.0 (7.0, 9.0) | 8.0 (7.0, 9.0) | 8.0 (7.0, 9.0) | 9.0 (8.0, 10.0) | 0.087 |
| 13.Postoperative glycaemic control | 135 | 8.0 (7.0, 9.0) | 8.0 (7.0, 9.0) | 8.0 (7.8, 9.0) | 8.0 (7.0, 9.0) | 0.7 |
| 14.Nasogastric (NG) intubation | 134 | 7.0 (5.0, 9.0) | 8.0 (5.8, 9.0) | 7.0 (3.5, 8.0) | 7.0 (5.5, 9.0) | 0.11 |
| 15.Fluid balance | 132 | 9.0 (8.0, 10.0) | 9.0 (8.2, 10.0) | 9.0 (8.0, 10.0) | 8.0 (7.0, 10.0) | 0.13 |
| 16.Peri-anastomotic drainage | 132 | 7.5 (5.0, 9.0) | 8.0 (7.0, 9.0) | 7.0 (5.0, 8.0) | 7.0 (4.5, 8.0) | 0.11 |
| 17.Use of somatostatin analogues | 135 | 4.0 (0.0, 6.0) | 5.5 (1.5, 7.0) | 3.0 (0.0, 5.2) | 4.0 (2.0, 6.0) | 0.2 |
| 18.Urinary drainage | 132 | 7.0 (5.0, 8.0) | 6.0 (5.0, 8.0) | 7.0 (5.0, 8.0) | 7.0 (5.0, 8.0) | 0.8 |
| 19.Stimulation of bowel movement | 131 | 7.0 (5.0, 8.5) | 7.0 (5.0, 9.0) | 6.0 (4.0, 8.0) | 7.0 (6.0, 9.0) | 0.13 |
| 20.Post-operative nutrition | 134 | 9.0 (7.0, 10.0) | 9.0 (8.0, 10.0) | 8.0 (7.0, 9.0) | 9.0 (8.0, 10.0) | 0.2 |
| 21.Post-operative mobilization | 133 | 9.0 (8.0, 10.0) | 9.0 (8.0, 10.0) | 9.0 (8.0, 10.0) | 9.0 (9.0, 10.0) | >0.9 |
| 22.Minimally invasive PD | 132 | 6.0 (3.0, 8.0) | 7.0 (5.0, 8.5) | 5.0 (2.0, 7.0) | 5.0 (3.0, 7.0) | 0.019 |

*Data are presented as median and (25^th^-75^th^) percentiles. P values are calculated* from Kruskal-Wallis test.

**Supplementary Table 3A: Challenges to the application of individual ERAS® components by sex (Abbreviations: ERAS – enhanced recovery after surgery; PD – pancreatoduodenectomy)**

|  | **N** | **Overall**, N = 140 | **Male**, N = 121 | **Female**, N = 19 | **p-value** |
| --- | --- | --- | --- | --- | --- |
| 1.Preoperative counselling | 137 | 7 (4.0, 9) | 7 (4.0, 9) | 7 (6.0, 9) | 0.2 |
| 2.Pre-habilitation | 132 | 8 (7.0, 9) | 8 (7.0, 9) | 7 (6.0, 8) | 0.3 |
| 3.Routine PBD | 134 | 5 (2.0, 7) | 5 (2.0, 7) | 6 (3.5, 7) | 0.080 |
| 4.Smoking cessation | 132 | 8 (6.0, 9) | 8 (6.0, 9) | 8 (7.0, 10) | 0.3 |
| 5.Pre-op nutritional intervention | 134 | 7 (5.0, 9) | 7 (5.0, 8) | 7 (5.0, 9) | 0.7 |
| 6.Carbohydrate loading | 133 | 6 (4.0, 8) | 6 (4.0, 8) | 7 (5.0, 8) | 0.5 |
| 7.Pre-anaesthetic medication | 131 | 6 (3.0, 8) | 5 (3.0, 8) | 7 (3.0, 9) | 0.6 |
| 8.Thrombotic prophylaxis | 129 | 6 (3.0, 8) | 6 (2.0, 8) | 6 (3.0, 8) | >0.9 |
| 9.Antimicrobial prophylaxis | 131 | 6 (2.0, 8) | 6 (2.0, 8) | 5 (2.2, 8) | 0.7 |
| 10.Analgesia | 129 | 7 (3.0, 9) | 7 (3.0, 8) | 7 (3.2, 9) | >0.9 |
| 11.PONV prophylaxis | 128 | 6 (3.0, 8) | 6 (3.0, 8) | 5 (3.0, 9) | >0.9 |
| 12.Avoiding hypothermia | 127 | 6 (3.0, 8) | 6 (3.0, 8) | 7 (3.0, 9) | >0.9 |
| 13.Postoperative glycaemic control | 130 | 6 (4.0, 8) | 6 (4.0, 8) | 6 (3.0, 7) | 0.4 |
| 14.Nasogastric (NG) intubation | 129 | 7 (3.0, 8) | 7 (3.0, 8) | 7 (4.5, 9) | 0.3 |
| 15.Fluid balance | 130 | 7 (5.0, 9) | 7 (5.0, 9) | 7 (6.2, 9) | 0.6 |
| 16.Peri-anastomotic drainage | 129 | 6 (4.0, 8) | 6 (3.0, 8) | 7 (6.0, 9) | 0.069 |
| 17.Use of somatostatin analogues | 128 | 3 (0.0, 6) | 3 (1.0, 6) | 5 (0.0, 6) | 0.7 |
| 18.Urinary drainage | 129 | 4 (2.0, 7) | 4 (2.0, 7) | 5 (2.2, 7) | 0.6 |
| 19.Stimulation of bowel movement | 129 | 5 (2.0, 8) | 5 (2.0, 8) | 6 (3.0, 8) | 0.5 |
| 20.Post-operative nutrition | 129 | 7 (5.0, 8) | 7 (4.5, 8) | 7 (5.2, 9) | >0.9 |
| 21.Post-operative mobilization | 129 | 8 (4.0, 9) | 8 (4.0, 9) | 7 (5.0, 9) | 0.9 |
| 22.Minimally invasive PD | 125 | 7 (5.0, 9) | 7 (4.0, 8) | 9 (7.0, 9) | 0.047 |

*Data are presented as median and (25^th^-75^th^) percentiles. P values are calculated from* Mann-Whitney U test.

**Supplementary Table 3B: Challenges to the application of individual ERAS® components by geographical area (Abbreviations: ERAS – enhanced recovery after surgery; PD – pancreatoduodenectomy)**

| **Characteristic** | **N** | **Overall**, N = 140 | **Europe**, N = 63 | **Asia/Oceania**, N = 42 | **North America**, N = 24 | **South America**, N = 11 | **p-value** |
| --- | --- | --- | --- | --- | --- | --- | --- |
| 1.Preoperative counselling | 137 | 7.0 (4.0, 9.0) | 6.0 (4.0, 8.0) | 8.0 (5.0, 10.0) | 5.0 (3.0, 8.0) | 6.0 (3.5, 8.5) | 0.033 |
| 2.Pre-habilitation | 132 | 8.0 (7.0, 9.0) | 8.0 (7.0, 9.0) | 8.0 (7.0, 9.0) | 8.0 (7.0, 9.0) | 6.0 (2.5, 7.5) | 0.2 |
| 3.Routine PBD | 134 | 5.0 (2.0, 7.0) | 5.0 (2.0, 7.0) | 5.0 (0.0, 7.0) | 5.0 (2.0, 6.2) | 6.0 (3.5, 7.0) | 0.8 |
| 4.Smoking cessation | 132 | 8.0 (6.0, 9.0) | 8.0 (5.5, 9.0) | 8.0 (7.0, 9.0) | 7.5 (6.0, 8.0) | 7.0 (7.0, 7.5) | 0.081 |
| 5.Pre-op nutritional intervention | 134 | 7.0 (5.0, 8.8) | 7.0 (5.0, 9.0) | 8.0 (6.0, 9.0) | 7.0 (5.0, 8.0) | 5.0 (3.0, 7.5) | 0.06 |
| 6.Carbohydrate loading | 133 | 6.0 (4.0, 8.0) | 6.0 (3.0, 8.0) | 7.0 (5.5, 9.0) | 6.0 (3.0, 7.2) | 5.0 (3.5, 6.5) | 0.013 |
| 7.Pre-anaesthetic medication | 131 | 6.0 (3.0, 8.0) | 5.0 (3.0, 8.0) | 7.0 (4.0, 9.0) | 4.0 (3.0, 8.0) | 5.0 (2.5, 8.5) | 0.4 |
| 8.Thrombotic prophylaxis | 129 | 6.0 (3.0, 8.0) | 5.0 (3.0, 8.0) | 7.0 (4.5, 9.0) | 4.5 (2.0, 6.2) | 4.0 (1.0, 6.5) | 0.10 |
| 9.Antimicrobial prophylaxis | 131 | 6.0 (2.0, 8.0) | 4.0 (2.0, 8.0) | 7.0 (5.0, 9.0) | 3.0 (2.0, 8.2) | 2.0 (1.0, 5.5) | 0.026 |
| 10.Analgesia | 129 | 7.0 (3.0, 9.0) | 7.0 (3.2, 8.0) | 8.0 (5.0, 9.2) | 4.0 (2.8, 7.0) | 5.0 (2.0, 8.5) | 0.071 |
| 11.PONV prophylaxis | 128 | 5.5 (3.0, 8.0) | 5.0 (3.0, 8.0) | 7.0 (5.0, 8.5) | 3.5 (2.8, 7.2) | 2.0 (2.0, 8.5) | 0.2 |
| 12.Avoiding hypothermia | 127 | 6.0 (3.0, 8.0) | 5.5 (3.0, 8.0) | 8.0 (4.5, 9.0) | 5.0 (3.0, 6.2) | 4.0 (0.5, 7.8) | 0.065 |
| 13.Postoperative glycaemic control | 130 | 6.0 (4.0, 8.0) | 6.0 (4.5, 8.0) | 7.0 (5.0, 9.0) | 5.5 (4.0, 8.0) | 3.0 (1.0, 7.0) | 0.10 |
| 14.Nasogastric (NG) intubation | 129 | 7.0 (3.0, 8.0) | 7.0 (3.0, 8.0) | 8.0 (5.0, 9.0) | 5.0 (2.0, 8.0) | 3.0 (1.0, 6.0) | 0.012 |
| 15.Fluid balance | 130 | 7.0 (5.0, 9.0) | 7.0 (6.0, 9.0) | 8.0 (6.0, 9.0) | 6.0 (4.0, 8.2) | 5.0 (1.0, 7.0) | 0.024 |
| 16.Peri-anastomotic drainage | 129 | 6.0 (4.0, 8.0) | 7.0 (4.0, 8.0) | 7.0 (3.8, 9.0) | 5.5 (3.8, 8.0) | 5.0 (1.0, 7.0) | 0.2 |
| 17.Use of somatostatin analogues | 128 | 3.0 (0.0, 6.0) | 3.0 (2.0, 6.0) | 3.5 (1.5, 6.2) | 2.0 (0.0, 5.0) | 5.0 (1.0, 5.5) | 0.6 |
| 18.Urinary drainage | 129 | 4.0 (2.0, 7.0) | 4.0 (2.2, 7.0) | 5.0 (3.0, 7.0) | 2.0 (2.0, 4.5) | 4.0 (1.0, 6.0) | 0.2 |
| 19.Stimulation of bowel movement | 129 | 5.0 (2.0, 8.0) | 6.0 (3.0, 8.0) | 6.0 (2.0, 7.5) | 4.0 (2.0, 6.0) | 5.0 (2.5, 7.5) | 0.5 |
| 20.Post-operative nutrition | 129 | 7.0 (5.0, 8.0) | 7.0 (5.0, 8.0) | 7.5 (6.5, 9.2) | 6.0 (3.8, 8.0) | 5.0 (1.0,7.0) | 0.045 |
| 21.Post-operative mobilization | 129 | 8.0 (4.0, 9.0) | 8.0 (4.0, 9.0) | 8.0 (5.0, 10.0) | 8.0 (3.0, 9.0) | 5.0 (1.0, 7.5) | 0.3 |
| 22.Minimally invasive PD | 125 | 7.0 (5.0, 9.0) | 7.0 (5.0, 8.0) | 6.5 (5.0, 8.8) | 7.0 (4.5, 9.0) | 7.0 (4.0, 9.5) | >0.9 |

*Data are presented as median and (25^th^-75^th^) percentiles. P values are calculated* from Kruskal-Wallis test.

**Supplementary Table 3C: Challenges to the application of individual ERAS® components by years in practice (Abbreviations: ERAS – enhanced recovery after surgery; PD – pancreatoduodenectomy)**

| **Characteristic** | **N** | **Overall**, N = 140 | **<10 Years**, N = 50 | **10-20 Years**, N = 54 | **>20 Years**, N = 36 | **p-value** |
| --- | --- | --- | --- | --- | --- | --- |
| 1.Preoperative counselling | 137 | 7.0 (4.0, 9.0) | 7.0 (3.8, 8.2) | 6.0 (4.0, 8.8) | 7.0 (5.0, 9.0) | 0.7 |
| 2.Pre-habilitation | 132 | 8.0 (7.0, 9.0) | 8.0 (7.0, 9.0) | 8.0 (6.0, 9.0) | 8.0 (5.5, 8.5) | 0.2 |
| 3.Routine PBD | 134 | 5.0 (2.0, 7.0) | 5.0 (2.0, 7.0) | 4.5 (2.0, 6.8) | 6.0 (4.0, 7.0) | 0.3 |
| 4.Smoking cessation | 132 | 8.0 (6.0, 9.0) | 8.0 (6.0, 9.0) | 8.0 (6.0, 8.0) | 7.5 (6.0, 8.8) | 0.4 |
| 5.Pre-op nutritional intervention | 134 | 7.0 (5.0, 8.8) | 7.0 (5.8, 9.0) | 7.0 (5.0, 8.0) | 7.0 (5.0, 9.0) | 0.4 |
| 6.Carbohydrate loading | 133 | 6.0 (4.0, 8.0) | 7.0 (4.8, 8.0) | 6.0 (3.0, 8.0) | 7.0 (5.0, 8.0) | 0.3 |
| 7.Pre-anaesthetic medication | 131 | 6.0 (3.0, 8.0) | 6.0 (3.0, 8.0) | 5.0 (3.0, 8.0) | 7.0 (3.0, 8.5) | 0.4 |
| 8.Thrombotic prophylaxis | 129 | 6.0 (3.0, 8.0) | 6.0 (2.0, 8.0) | 5.0 (3.0, 7.0) | 7.0 (2.2, 9.0) | 0.3 |
| 9.Antimicrobial prophylaxis | 131 | 6.0 (2.0, 8.0) | 6.0 (3.0, 8.0) | 4.5 (2.0, 8.0) | 7.0 (2.5, 9.0) | 0.5 |
| 10.Analgesia | 129 | 7.0 (3.0, 9.0) | 7.0 (3.0, 8.2) | 5.0 (3.0, 8.0) | 7.0 (3.5, 9.0) | 0.3 |
| 11.PONV prophylaxis | 128 | 5.5 (3.0, 8.0) | 6.0 (2.0, 8.0) | 5.0 (3.0, 8.0) | 7.0 (2.2, 9.0) | 0.4 |
| 12.Avoiding hypothermia | 127 | 6.0 (3.0, 8.0) | 6.0 (2.8, 8.0) | 5.0 (3.0, 7.5) | 7.0 (3.8, 9.0) | 0.3 |
| 13.Postoperative glycaemic control | 130 | 6.0 (4.0, 8.0) | 6.0 (3.0, 8.0) | 6.0 (5.0, 8.0) | 8.0 (4.5, 9.0) | 0.2 |
| 14.Nasogastric (NG) intubation | 129 | 7.0 (3.0, 8.0) | 7.0 (3.0, 9.0) | 6.0 (3.0, 8.5) | 6.5 (2.2, 8.0) | 0.6 |
| 15.Fluid balance | 130 | 7.0 (5.0, 9.0) | 7.0 (6.0, 9.0) | 7.0 (4.5, 9.0) | 7.0 (5.0, 9.0) | 0.6 |
| 16.Peri-anastomotic drainage | 129 | 6.0 (4.0, 8.0) | 7.0 (5.0, 8.0) | 6.0 (2.5, 8.0) | 5.5 (4.2, 7.0) | 0.2 |
| 17.Use of somatostatin analogues | 128 | 3.0 (0.0, 6.0) | 3.0 (0.0, 7.0) | 3.0 (2.0, 5.5) | 3.0 (0.5, 5.8) | >0.9 |
| 18.Urinary drainage | 129 | 4.0 (2.0, 7.0) | 5.0 (2.0, 7.0) | 3.0 (2.0, 7.0) | 5.0 (2.0, 7.0) | 0.3 |
| 19.Stimulation of bowel movement | 129 | 5.0 (2.0, 8.0) | 6.0 (2.0, 8.0) | 5.0 (3.0, 8.0) | 5.0 (3.0, 7.0) | 0.9 |
| 20.Post-operative nutrition | 129 | 7.0 (5.0, 8.0) | 7.0 (5.0, 9.0) | 7.0 (4.5, 8.0) | 6.5 (3.2, 8.0) | 0.4 |
| 21.Post-operative mobilization | 129 | 8.0 (4.0, 9.0) | 8.0 (5.0, 9.0) | 7.0 (2.5, 9.0) | 8.0 (5.0, 9.0) | 0.4 |
| 22.Minimally invasive PD | 125 | 7.0 (5.0, 9.0) | 7.0 (5.0, 9.0) | 7.0 (3.8, 9.0) | 6.0 (2.2, 8.0) | 0.12 |

*Data are presented as median and (25^th^-75^th^) percentiles. P values are calculated* from Kruskal-Wallis test.

**Supplementary Table 3D: Challenges to the application of individual ERAS® components by case-mix (Abbreviations: ERAS – enhanced recovery after surgery; HPB – hepato-pancreato-biliary; PD – pancreatoduodenectomy)**

| **Characteristic** | **N** | **Overall**, N = 140 | **HPB Surgery**, N = 96 | **General Surgery**, N = 25 | **Pancreatic Surgery**, N = 11 | **Others**, N = 8 | **p-value** |
| --- | --- | --- | --- | --- | --- | --- | --- |
| 1.Preoperative counselling | 137 | 7.0 (4.0, 9.0) | 7.0 (5.0, 9.0) | 6.5 (3.0, 8.0) | 5.5 (3.2, 7.8) | 7.5 (3.0, 10.0) | 0.6 |
| 2.Pre-habilitation | 132 | 8.0 (7.0, 9.0) | 8.0 (7.0, 9.0) | 8.0 (6.0, 9.0) | 8.0 (7.0, 9.0) | 7.0 (5.5, 8.5) | 0.9 |
| 3.Routine PBD | 134 | 5.0 (2.0, 7.0) | 5.0 (2.0, 7.0) | 5.0 (2.8, 7.0) | 4.0 (2.2, 5.0) | 7.0 (4.5, 7.5) | 0.3 |
| 4.Smoking cessation | 132 | 8.0 (6.0, 9.0) | 8.0 (6.0, 9.0) | 8.0 (6.0, 9.0) | 7.5 (5.8, 8.0) | 9.5 (7.8, 10.0) | 0.3 |
| 5.Pre-op nutritional intervention | 134 | 7.0 (5.0, 8.8) | 7.0 (5.0, 9.0) | 7.5 (6.0, 8.0) | 8.0 (7.0, 8.0) | 7.5 (5.0, 9.2) | 0.8 |
| 6.Carbohydrate loading | 133 | 6.0 (4.0, 8.0) | 6.0 (4.0, 8.0) | 7.0 (3.0, 8.0) | 5.0 (4.8, 7.0) | 4.5 (2.8, 7.5) | 0.7 |
| 7.Pre-anaesthetic medication | 131 | 6.0 (3.0, 8.0) | 6.0 (3.0, 8.0) | 5.5 (2.8, 8.0) | 4.0 (2.8, 7.5) | 3.0 (3.0, 4.0) | 0.5 |
| 8.Thrombotic prophylaxis | 129 | 6.0 (3.0, 8.0) | 6.0 (3.0, 8.8) | 4.5 (2.0, 8.0) | 3.0 (2.0, 5.5) | 3.0 (2.8, 5.5) | 0.3 |
| 9.Antimicrobial prophylaxis | 131 | 6.0 (2.0, 8.0) | 6.0 (3.0, 9.0) | 4.0 (2.0, 8.0) | 3.5 (2.0, 6.5) | 3.5 (2.0, 8.5) | 0.7 |
| 10.Analgesia | 129 | 7.0 (3.0, 9.0) | 7.0 (3.0, 9.0) | 7.0 (3.0, 8.2) | 5.0 (3.5, 7.0) | 6.0 (4.0, 8.5) | 0.8 |
| 11.PONV prophylaxis | 128 | 5.5 (3.0, 8.0) | 6.0 (3.0, 8.0) | 5.0 (2.0, 8.0) | 3.0 (2.0, 7.2) | 3.5 (2.8, 4.5) | 0.4 |
| 12.Avoiding hypothermia | 127 | 6.0 (3.0, 8.0) | 6.0 (3.0, 8.0) | 4.0 (2.0, 8.0) | 3.0 (2.0, 6.5) | 5.0 (3.0, 9.0) | 0.3 |
| 13.Postoperative glycaemic control | 130 | 6.0 (4.0, 8.0) | 6.0 (4.0, 8.0) | 7.0 (3.0, 8.2) | 5.5 (3.5, 8.0) | 6.0 (5.0, 7.5) | >0.9 |
| 14.Nasogastric (NG) intubation | 129 | 7.0 (3.0, 8.0) | 6.0 (3.0, 8.0) | 7.5 (2.8, 9.0) | 2.0 (2.0, 5.5) | 9.0 (5.8, 9.2) | 0.14 |
| 15.Fluid balance | 130 | 7.0 (5.0, 9.0) | 7.0 (5.0, 9.0) | 8.0 (6.8, 9.0) | 7.5 (6.8, 8.2) | 6.5 (3.8, 7.2) | 0.5 |
| 16.Peri-anastomotic drainage | 129 | 6.0 (4.0, 8.0) | 6.0 (3.0, 8.0) | 7.0 (5.8, 8.2) | 5.0 (4.5, 8.0) | 7.0 (6.0, 9.2) | 0.2 |
| 17.Use of somatostatin analogues | 128 | 3.0 (0.0, 6.0) | 3.0 (0.0, 6.0) | 5.0 (3.8, 6.2) | 2.0 (0.0, 4.0) | 5.0 (2.0, 7.2) | 0.040 |
| 18.Urinary drainage | 129 | 4.0 (2.0, 7.0) | 4.0 (2.0, 7.0) | 5.0 (3.0, 7.0) | 2.0 (2.0, 5.0) | 3.0 (2.0, 7.5) | 0.7 |
| 19.Stimulation of bowel movement | 129 | 5.0 (2.0, 8.0) | 5.0 (3.0, 8.0) | 6.0 (2.0, 8.0) | 4.0 (2.0, 7.2) | 4.0 (2.8, 4.2) | 0.7 |
| 20.Post-operative nutrition | 129 | 7.0 (5.0, 8.0) | 7.0 (5.0, 8.0) | 7.5 (4.8, 8.2) | 7.0 (4.5, 8.0) | 4.5 (3.0, 6.8) | 0.7 |
| 21.Post-operative mobilization | 129 | 8.0 (4.0, 9.0) | 8.0 (5.0, 9.0) | 8.0 (5.0, 9.0) | 8.0 (3.8, 9.2) | 4.0 (2.8, 8.2) | 0.7 |
| 22.Minimally invasive PD | 125 | 7.0 (5.0, 9.0) | 7.0 (4.0, 8.0) | 8.0 (6.8, 9.0) | 7.0 (6.0, 8.5) | 4.0 (1.0, 6.0) | 0.051 |

*Data are presented as median and (25^th^-75^th^) percentiles. P values are calculated* from Kruskal-Wallis test.

**Supplementary Table 3E: Challenges to the application of individual ERAS® components by annual PD volume (Abbreviations: ERAS – enhanced recovery after surgery; PD – pancreatoduodenectomy)**

| **Characteristic** | **N** | **Overall**, N = 140 | **<20**, N = 33 | **20-50**, N = 57 | **>50**, N = 50 | **p-value** |
| --- | --- | --- | --- | --- | --- | --- |
| 1.Preoperative counselling | 136 | 9.0 (7.0, 10.0) | 9.0 (7.0, 10.0) | 8.5 (8.0, 10.0) | 9.0 (7.0, 9.0) | 0.9 |
| 2.Pre-habilitation | 135 | 8.0 (7.0, 9.0) | 8.5 (8.0, 10.0) | 8.0 (6.0, 9.8) | 8.0 (7.0, 9.0) | 0.2 |
| 3.Routine PBD | 136 | 5.0 (2.0, 6.0) | 5.0 (2.8, 6.0) | 5.0 (2.0, 6.0) | 5.0 (0.0, 7.0) | 0.6 |
| 4.Smoking cessation | 135 | 8.0 (6.0, 9.0) | 9.0 (7.8, 9.0) | 7.0 (6.0, 9.0) | 8.0 (6.0, 8.0) | 0.023 |
| 5.Pre-op nutritional intervention | 136 | 9.0 (7.0, 9.0) | 9.0 (8.0, 10.0) | 8.0 (7.0, 9.0) | 8.0 (6.0, 9.0) | 0.007 |
| 6.Carbohydrate loading | 136 | 7.0 (6.0, 8.0) | 7.0 (7.0, 9.0) | 7.0 (6.0, 8.0) | 7.0 (5.0, 8.0) | 0.15 |
| 7.Pre-anaesthetic medication | 134 | 7.0 (5.0, 8.8) | 8.0 (5.5, 9.0) | 7.0 (6.0, 9.0) | 7.0 (5.0, 8.0) | 0.7 |
| 8.Thrombotic prophylaxis | 134 | 8.0 (7.0, 9.0) | 9.0 (7.0, 10.0) | 8.0 (7.0, 9.0) | 8.0 (7.0, 9.0) | 0.5 |
| 9.Antimicrobial prophylaxis | 135 | 8.0 (7.0, 9.0) | 9.0 (8.0, 10.0) | 8.0 (7.0, 9.5) | 8.0 (7.0, 9.0) | 0.064 |
| 10.Analgesia | 134 | 9.0 (8.0, 10.0) | 9.5 (9.0, 10.0) | 9.0 (8.0, 10.0) | 8.0 (8.0, 9.0) | 0.005 |
| 11.PONV prophylaxis | 135 | 9.0 (8.0, 9.0) | 9.0 (8.0, 10.0) | 8.0 (7.2, 9.0) | 8.0 (8.0, 9.0) | 0.3 |
| 12.Avoiding hypothermia | 134 | 8.0 (7.0, 9.0) | 9.0 (8.0, 10.0) | 8.0 (7.0, 9.0) | 8.0 (7.0, 9.0) | 0.005 |
| 13.Postoperative glycaemic control | 135 | 8.0 (7.0, 9.0) | 9.0 (8.8, 10.0) | 8.0 (7.0, 9.0) | 8.0 (7.0, 9.0) | 0.001 |
| 14.Nasogastric (NG) intubation | 134 | 7.0 (5.0, 9.0) | 6.5 (5.0, 9.0) | 7.0 (6.0, 9.0) | 7.0 (5.0, 8.0) | 0.8 |
| 15.Fluid balance | 132 | 9.0 (8.0, 10.0) | 9.0 (8.0, 10.0) | 9.0 (8.0, 10.0) | 9.0 (8.0, 10.0) | 0.7 |
| 16.Peri-anastomotic drainage | 132 | 7.5 (5.0, 9.0) | 8.0 (6.0, 9.0) | 7.0 (4.8, 9.0) | 7.0 (5.8, 8.0) | 0.3 |
| 17.Use of somatostatin analogues | 135 | 4.0 (0.0, 6.0) | 5.0 (2.8, 6.2) | 4.0 (0.0, 6.0) | 4.5 (0.0, 6.2) | 0.4 |
| 18.Urinary drainage | 132 | 7.0 (5.0, 8.0) | 7.5 (5.8, 9.0) | 6.0 (3.0, 8.0) | 7.0 (5.0, 8.0) | 0.054 |
| 19.Stimulation of bowel movement | 131 | 7.0 (5.0, 8.5) | 8.0 (5.8, 9.2) | 7.0 (5.0, 9.0) | 6.0 (4.5, 7.5) | 0.030 |
| 20.Post-operative nutrition | 134 | 9.0 (7.0, 10.0) | 9.0 (8.0, 10.0) | 9.0 (8.0, 9.8) | 8.0 (7.0, 9.0) | 0.069 |
| 21.Post-operative mobilization | 133 | 9.0 (8.0, 10.0) | 9.0 (9.0, 10.0) | 9.0 (9.0, 10.0) | 9.0 (8.0, 9.0) | 0.015 |
| 22.Minimally invasive PD | 132 | 6.0 (3.0, 8.0) | 7.0 (5.0, 9.0) | 5.0 (3.0, 7.0) | 5.0 (2.0, 8.0) | 0.10 |

*Data are presented as median and (25^th^-75^th^) percentiles. P values are calculated* from Kruskal-Wallis test.

**Supplementary Table 3F: Challenges to the application of individual ERAS® components by unit’s PD patient care routine care practice (Abbreviations: ERAS – enhanced recovery after surgery; PD – pancreatoduodenectomy)**

| **Characteristic** | **N** | **Overall**, N = 140 | **ERAS® Pathways**, N = 45 | **Post-PD CP to Enhance Recovery**, N = 53 | **Management at Surgeon’s Discretion**, N = 42 | **p-value** |
| --- | --- | --- | --- | --- | --- | --- |
| 1.Preoperative counselling | 136 | 9.0 (7.0, 10.0) | 9.0 (7.5, 10.0) | 8.0 (7.0, 10.0) | 8.5 (8.0, 10.0) | 0.8 |
| 2.Pre-habilitation | 135 | 8.0 (7.0, 9.0) | 8.0 (6.8, 9.0) | 8.0 (7.0, 9.5) | 8.5 (7.8, 10.0) | 0.4 |
| 3.Routine PBD | 136 | 5.0 (2.0, 6.0) | 5.0 (3.8, 7.2) | 4.0 (1.5, 6.0) | 5.0 (0.0, 6.2) | 0.2 |
| 4.Smoking cessation | 135 | 8.0 (6.0, 9.0) | 7.0 (6.0, 9.0) | 8.0 (5.5, 9.0) | 8.0 (7.0, 9.0) | 0.3 |
| 5.Pre-op nutritional intervention | 136 | 9.0 (7.0, 9.0) | 9.0 (7.0, 9.2) | 8.5 (7.8, 9.0) | 8.0 (7.0, 9.2) | >0.9 |
| 6.Carbohydrate loading | 136 | 7.0 (6.0, 8.0) | 7.0 (6.0, 9.0) | 7.0 (5.0, 8.0) | 7.0 (6.0, 8.0) | 0.9 |
| 7.Pre-anaesthetic medication | 134 | 7.0 (5.0, 8.8) | 7.0 (5.5, 9.0) | 7.0 (5.0, 8.0) | 7.0 (5.0, 8.0) | >0.9 |
| 8.Thrombotic prophylaxis | 134 | 8.0 (7.0, 9.0) | 8.0 (7.0, 9.0) | 9.0 (7.0, 9.0) | 8.0 (7.0, 9.0) | 0.7 |
| 9.Antimicrobial prophylaxis | 135 | 8.0 (7.0, 9.0) | 8.0 (7.0, 9.0) | 8.5 (7.0, 10.0) | 9.0 (7.5, 9.0) | 0.4 |
| 10.Analgesia | 134 | 9.0 (8.0, 10.0) | 9.0 (8.0, 10.0) | 9.0 (8.0, 10.0) | 9.0 (8.0, 10.0) | 0.3 |
| 11.PONV prophylaxis | 135 | 9.0 (8.0, 9.0) | 8.0 (8.0, 9.5) | 8.0 (7.0, 9.0) | 9.0 (8.0, 10.0) | 0.2 |
| 12.Avoiding hypothermia | 134 | 8.0 (7.0, 9.0) | 8.0 (7.0, 9.0) | 8.0 (7.0, 9.0) | 9.0 (8.0, 10.0) | 0.087 |
| 13.Postoperative glycaemic control | 135 | 8.0 (7.0, 9.0) | 8.0 (7.0, 9.0) | 8.0 (7.8, 9.0) | 8.0 (7.0, 9.0) | 0.7 |
| 14.Nasogastric (NG) intubation | 134 | 7.0 (5.0, 9.0) | 8.0 (5.8, 9.0) | 7.0 (3.5, 8.0) | 7.0 (5.5, 9.0) | 0.11 |
| 15.Fluid balance | 132 | 9.0 (8.0, 10.0) | 9.0 (8.2, 10.0) | 9.0 (8.0, 10.0) | 8.0 (7.0, 10.0) | 0.13 |
| 16.Peri-anastomotic drainage | 132 | 7.5 (5.0, 9.0) | 8.0 (7.0, 9.0) | 7.0 (5.0, 8.0) | 7.0 (4.5, 8.0) | 0.11 |
| 17.Use of somatostatin analogues | 135 | 4.0 (0.0, 6.0) | 5.5 (1.5, 7.0) | 3.0 (0.0, 5.2) | 4.0 (2.0, 6.0) | 0.2 |
| 18.Urinary drainage | 132 | 7.0 (5.0, 8.0) | 6.0 (5.0, 8.0) | 7.0 (5.0, 8.0) | 7.0 (5.0, 8.0) | 0.8 |
| 19.Stimulation of bowel movement | 131 | 7.0 (5.0, 8.5) | 7.0 (5.0, 9.0) | 6.0 (4.0, 8.0) | 7.0 (6.0, 9.0) | 0.13 |
| 20.Post-operative nutrition | 134 | 9.0 (7.0, 10.0) | 9.0 (8.0, 10.0) | 8.0 (7.0, 9.0) | 9.0 (8.0, 10.0) | 0.2 |
| 21.Post-operative mobilization | 133 | 9.0 (8.0, 10.0) | 9.0 (8.0, 10.0) | 9.0 (8.0, 10.0) | 9.0 (9.0, 10.0) | >0.9 |
| 22.Minimally invasive PD | 132 | 6.0 (3.0, 8.0) | 7.0 (5.0, 8.5) | 5.0 (2.0, 7.0) | 5.0 (3.0, 7.0) | 0.019 |

*Data are presented as median and (25^th^-75^th^) percentiles. P values are calculated* from Kruskal-Wallis test.

**Supplementary Table 4A: Facilitators to implementation and sustainability of peri-PD ERAS® pathways by sex (Abbreviations: ERAS – enhanced recovery after surgery; PD – pancreatoduodenectomy)**

| **Characteristic** | **N** | **Overall**, N = 140 | **Male**, N = 121 | **Female**, N = 19 | **p-value** |
| --- | --- | --- | --- | --- | --- |
| 1.Multidisciplinary and coordination between the different members | 134 | 9.0 (8.0, 10.0) | 9.0 (8.0, 10.0) | 10.0 (9.0, 10.0) | 0.12 |
| 2.Patient’s empowerment | 133 | 8.0 (7.0, 9.0) | 8.0 (7.0, 9.0) | 9.0 (7.0, 10.0) | 0.13 |
| 3.Regular audit and continuous improvement process | 132 | 8.0 (7.0, 10.0) | 8.0 (7.0, 10.0) | 9.0 (7.2, 9.8) | 0.5 |
| 4.ERAS dedicated nurse | 130 | 8.0 (7.0, 10.0) | 8.0 (7.0, 10.0) | 9.0 (8.0, 10.0) | 0.12 |
| 5.Clear discharge criteria | 131 | 8.0 (7.0, 9.5) | 8.0 (7.0, 9.0) | 9.5 (8.2, 10.0) | 0.011 |

*Data are presented as median and (25^th^-75^th^) percentiles. P values are calculated from* Mann-Whitney U test.

**Supplementary Table 4B: Facilitators to implementation and sustainability of peri-PD ERAS® pathways by geographical area (Abbreviations: ERAS – enhanced recovery after surgery; PD – pancreatoduodenectomy)**

| **Characteristic** | **N** | **Overall**, N = 140 | **Europe**, N = 63 | **Asia/Oceania**, N = 42 | **North America**, N = 24 | **South America**, N = 11 | **p-value** |
| --- | --- | --- | --- | --- | --- | --- | --- |
| 1.Multidisciplinary and coordination between the different members | 134 | 9.0 (8.0, 10.0) | 9.0 (8.0, 10.0) | 9.0 (8.5, 10.0) | 8.0 (7.0, 9.0) | 10.0 (10.0, 10.0) | <0.001 |
| 2.Patient’s empowerment | 133 | 8.0 (7.0, 9.0) | 8.0 (7.0, 9.0) | 9.0 (8.0, 10.0) | 7.5 (5.0, 8.0) | 8.0 (7.0, 9.5) | 0.039 |
| 3.Regular audit and continuous improvement process | 132 | 8.0 (7.0, 10.0) | 8.0 (7.0, 9.0) | 9.0 (8.0, 10.0) | 8.0 (6.8, 8.2) | 9.0 (7.5, 10.0) | 0.11 |
| 4.ERAS dedicated nurse | 130 | 8.0 (7.0, 10.0) | 8.5 (7.0, 10.0) | 8.0 (7.0, 10.0) | 6.5 (5.0, 8.0) | 10.0 (7.5, 10.0) | 0.013 |
| 5.Clear discharge criteria | 131 | 8.0 (7.0, 9.5) | 8.0 (7.0, 9.0) | 9.0 (8.0, 10.0) | 8.0 (6.5, 9.0) | 10.0 (9.5, 10.0) | 0.002 |

*Data are presented as median and (25^th^-75^th^) percentiles. P values are calculated* from Kruskal-Wallis test.

**Supplementary Table 4C: Facilitators to implementation and sustainability of peri-PD ERAS® pathways by years in practice (Abbreviations: ERAS – enhanced recovery after surgery; PD – pancreatoduodenectomy)**

| **Characteristic** | **N** | **Overall**, N = 140 | **<10 Years**, N = 50 | **10-20 Years**, N = 54 | **>20 Years**, N = 36 | **p-value** |
| --- | --- | --- | --- | --- | --- | --- |
| 1.Multidisciplinary and coordination between the different members | 134 | 9.0 (8.0, 10.0) | 9.0 (8.0, 10.0) | 9.0 (8.0, 10.0) | 10.0 (9.0, 10.0) | 0.030 |
| 2.Patient’s empowerment | 133 | 8.0 (7.0, 9.0) | 8.0 (7.0, 9.0) | 8.0 (7.0, 9.0) | 8.0 (7.0, 9.0) | 0.7 |
| 3.Regular audit and continuous improvement process | 132 | 8.0 (7.0, 10.0) | 9.0 (7.0, 9.0) | 8.0 (5.8, 10.0) | 9.0 (7.0, 10.0) | 0.2 |
| 4.ERAS dedicated nurse | 130 | 8.0 (7.0, 10.0) | 8.0 (7.0, 10.0) | 8.0 (5.5, 10.0) | 8.0 (7.0, 10.0) | 0.6 |
| 5.Clear discharge criteria | 131 | 8.0 (7.0, 9.5) | 8.0 (7.0, 9.0) | 8.0 (7.0, 10.0) | 9.0 (8.0, 10.0) | 0.2 |

*Data are presented as median and (25^th^-75^th^) percentiles. P values are calculated* from Kruskal-Wallis test.

**Supplementary Table 4D: Facilitators to implementation and sustainability of peri-PD ERAS® pathways by case-mix (Abbreviations: ERAS – enhanced recovery after surgery; HPB – hepato-pancreato-biliary; PD – pancreatoduodenectomy)**

| **Characteristic** | **N** | **Overall**, N = 140 | **HPB Surgery**,  N = 96 | **General Surgery**,  N = 25 | **Pancreatic Surgery**,  N = 11 | **Others,** N=8 | **p-value** |
| --- | --- | --- | --- | --- | --- | --- | --- |
| 1.Multidisciplinary and coordination between the different members | 134 | 9.0 (8.0, 10.0) | 9.0 (8.0, 10.0) | 9.0 (9.0, 10.0) | 9.0 (8.0, 10.0) | 9.0 (7.0, 9.2) | 0.6 |
| 2.Patient’s empowerment | 133 | 8.0 (7.0, 9.0) | 8.0 (7.0, 9.0) | 8.0 (7.0, 9.0) | 7.5 (7.0, 8.0) | 7.5 (5.8, 9.2) | 0.7 |
| 3.Regular audit and continuous improvement process | 132 | 8.0 (7.0, 10.0) | 8.0 (7.0, 10.0) | 9.0 (8.0, 10.0) | 7.5 (6.2, 9.0) | 7.5 (5.8, 8.0) | 0.091 |
| 4.ERAS dedicated nurse | 130 | 8.0 (7.0, 10.0) | 8.0 (6.0, 10.0) | 9.0 (8.0, 10.0) | 8.0 (6.2, 8.8) | 8.0 (7.0, 9.0) | 0.10 |
| 5.Clear discharge criteria | 131 | 8.0 (7.0, 9.5) | 8.0 (8.0, 10.0) | 8.0 (7.0, 10.0) | 7.5 (7.0, 8.0) | 8.0 (5.0, 9.0) | 0.3 |

*Data are presented as median and (25^th^-75^th^) percentiles. P values are calculated* from Kruskal-Wallis test.

**Supplementary Table 4E: Facilitators to implementation and sustainability of peri-PD ERAS® pathways by annual PD volume (Abbreviations: ERAS – enhanced recovery after surgery; PD – pancreatoduodenectomy)**

| **Characteristic** | **N** | **Overall**, N = 140 | **<20**, N = 33 | **20-50**, N = 57 | **>50**, N = 50 | **p-value** |
| --- | --- | --- | --- | --- | --- | --- |
| 1.Multidisciplinary and coordination between the different members | 134 | 9.0 (8.0, 10.0) | 10.0 (9.0, 10.0) | 9.0 (7.2, 10.0) | 9.0 (8.0, 10.0) | 0.008 |
| 2.Patient’s empowerment | 133 | 8.0 (7.0, 9.0) | 9.0 (8.0, 10.0) | 8.0 (7.0, 9.0) | 8.0 (7.0, 9.0) | 0.043 |
| 3.Regular audit and continuous improvement process | 132 | 8.0 (7.0, 10.0) | 9.0 (8.0, 10.0) | 8.0 (6.0, 10.0) | 8.0 (7.0, 9.0) | 0.008 |
| 4.ERAS dedicated nurse | 130 | 8.0 (7.0, 10.0) | 9.5 (8.0, 10.0) | 8.0 (7.0, 10.0) | 7.5 (6.0, 9.0) | 0.003 |
| 5.Clear discharge criteria | 131 | 8.0 (7.0, 9.5) | 9.5 (8.2, 10.0) | 8.0 (8.0, 9.5) | 8.0 (6.2, 9.0) | <0.001 |

*Data are presented as median and (25^th^-75^th^) percentiles. P values are calculated* from Kruskal-Wallis test.

**Supplementary Table 4F: Facilitators to implementation and sustainability of peri-PD ERAS® pathways by unit’s PD patient care routine care practice (Abbreviations: ERAS – enhanced recovery after surgery; PD – pancreatoduodenectomy)**

| **Characteristic** | **N** | **Overall**, N = 140 | **ERAS® Pathways**, N = 45 | **Post-PD CP to Enhance Recovery**, N = 53 | **Management at Surgeon’s Discretion**, N = 42 | **p-value** |
| --- | --- | --- | --- | --- | --- | --- |
| 1.Multidisciplinary and coordination between the different members | 134 | 9.0 (8.0, 10.0) | 9.0 (8.0, 10.0) | 9.0 (8.0, 10.0) | 9.0 (8.0, 10.0) | 0.8 |
| 2.Patient’s empowerment | 133 | 8.0 (7.0, 9.0) | 8.0 (7.0, 9.0) | 8.0 (7.0, 9.0) | 8.0 (7.0, 9.8) | 0.6 |
| 3.Regular audit and continuous improvement process | 132 | 8.0 (7.0, 10.0) | 9.0 (8.0, 9.0) | 8.0 (6.0, 9.2) | 8.5 (7.0, 10.0) | 0.2 |
| 4.ERAS dedicated nurse | 130 | 8.0 (7.0, 10.0) | 9.0 (7.0, 10.0) | 8.0 (5.2, 9.0) | 8.0 (7.0, 10.0) | 0.11 |
| 5.Clear discharge criteria | 131 | 8.0 (7.0, 9.5) | 8.0 (7.0, 9.0) | 8.0 (7.0, 9.0) | 9.0 (8.0, 10.0) | 0.003 |

*Data are presented as median and (25^th^-75^th^) percentiles. P values are calculated* from Kruskal-Wallis test.

**Supplementary Table 5A: Barriers to implementation and sustainability of peri-PD ERAS® pathways by sex (Abbreviations: ERAS – enhanced recovery after surgery; PD – pancreatoduodenectomy)**

| **Characteristic** | **N** | **Overall**, N = 140 | **Male**, N = 121 | **Female**, N = 19 | **p-value** |
| --- | --- | --- | --- | --- | --- |
| 1.Reluctance to change from the healthcare practitioners | 136 | 8.0 (6.0, 9.0) | 8.0 (6.0, 9.0) | 8.0 (5.5, 9.0) | 0.4 |
| 2.Low patient’s involvement and motivation | 136 | 6.0 (4.0, 8.0) | 6.0 (4.2, 8.0) | 6.0 (4.2, 8.0) | 0.8 |
| 3.Difficulty in collaboration between members of the multidisciplinary team | 134 | 7.0 (6.0, 8.8) | 7.0 (6.0, 8.2) | 7.5 (5.0, 8.8) | >0.9 |
| 4.Initial time and money investment | 135 | 7.0 (5.0, 9.0) | 7.0 (5.0, 9.0) | 7.5 (5.0, 9.0) | >0.9 |
| 5.Data collection and audit | 133 | 8.0 (6.0, 9.0) | 8.0 (6.0, 9.0) | 6.5 (5.0, 8.0) | 0.12 |
| 6.Lack of administrative support | 132 | 8.0 (6.0, 9.0) | 8.0 (6.0, 9.0) | 8.0 (7.0, 9.0) | 0.7 |
| 7.Recruitment of an ERAS-dedicated nurse | 132 | 8.0 (6.0, 9.0) | 8.0 (6.0, 9.0) | 7.0 (5.0, 9.0) | 0.7 |

*Data are presented as median and (25^th^-75^th^) percentiles. P values are calculated* from Kruskal-Wallis test.

**Supplementary Table 5B: Barriers to implementation and sustainability of peri-PD ERAS® pathways by geographical area (Abbreviations: ERAS – enhanced recovery after surgery; PD – pancreatoduodenectomy)**

| **Characteristic** | **N** | **Overall**, N = 140 | **Europe**, N = 63 | **Asia/Oceania**, N = 42 | **North America**, N = 24 | **South America**, N = 11 | **p-value** |
| --- | --- | --- | --- | --- | --- | --- | --- |
| 1.Reluctance to change from the healthcare practitioners | 136 | 8.0 (6.0, 9.0) | 8.0 (7.0, 8.0) | 8.0 (7.0, 10.0) | 6.0 (5.0, 8.0) | 9.0 (8.0, 10.0) | 0.005 |
| 2.Low patient’s involvement and motivation | 136 | 6.0 (4.0, 8.0) | 6.0 (4.2, 8.0) | 7.0 (5.0, 8.2) | 5.0 (4.0, 7.0) | 6.0 (3.5, 8.5) | 0.2 |
| 3.Difficulty in collaboration between members of the multidisciplinary team | 134 | 7.0 (6.0, 8.8) | 7.0 (6.0, 8.2) | 7.0 (5.8, 9.0) | 6.0 (5.0, 7.5) | 8.0 (6.5, 9.0) | 0.3 |
| 4.Initial time and money investment | 135 | 7.0 (5.0, 9.0) | 7.0 (5.0, 8.0) | 7.0 (4.8, 9.0) | 7.0 (5.0, 8.5) | 9.0 (7.0, 10.0) | 0.4 |
| 5.Data collection and audit | 133 | 8.0 (6.0, 9.0) | 7.0 (6.0, 8.0) | 8.0 (5.0, 10.0) | 7.0 (7.0, 8.0) | 8.0 (7.0, 9.5) | 0.3 |
| 6.Lack of administrative support | 132 | 8.0 (6.0, 9.0) | 7.0 (6.0, 9.0) | 7.5 (5.0, 9.0) | 8.0 (6.0, 9.0) | 10.0 (8.5, 10.0) | 0.040 |
| 7.Recruitment of an ERAS-dedicated nurse | 132 | 8.0 (6.0, 9.0) | 7.5 (5.0, 9.0) | 8.0 (6.0, 10.0) | 8.0 (6.0, 9.0) | 10.0 (5.0, 10.0) | 0.3 |

*Data are presented as median and (25^th^-75^th^) percentiles. P values are calculated* from Kruskal-Wallis test.

**Supplementary Table 5C: Barriers to implementation and sustainability of peri-PD ERAS® pathways by years in practice (Abbreviations: ERAS – enhanced recovery after surgery; PD – pancreatoduodenectomy)**

| **Characteristic** | **N** | **Overall**, N = 140 | **<10 Years**, N = 50 | **10-20 Years**, N = 54 | **>20 Years**, N = 36 | **p-value** |
| --- | --- | --- | --- | --- | --- | --- |
| 1.Reluctance to change from the healthcare practitioners | 136 | 8.0 (6.0, 9.0) | 8.0 (7.0, 10.0) | 8.0 (6.0, 9.0) | 8.0 (7.0, 9.0) | 0.6 |
| 2.Low patient’s involvement and motivation | 136 | 6.0 (4.0, 8.0) | 6.0 (4.8, 8.0) | 6.0 (4.0, 8.0) | 7.0 (5.0, 8.0) | 0.2 |
| 3.Difficulty in collaboration between members of the multidisciplinary team | 134 | 7.0 (6.0, 8.8) | 7.0 (6.0, 9.0) | 7.0 (5.0, 8.0) | 8.0 (6.5, 9.0) | 0.5 |
| 4.Initial time and money investment | 135 | 7.0 (5.0, 9.0) | 7.5 (5.8, 9.0) | 7.0 (4.0, 8.2) | 7.0 (5.0, 9.0) | 0.4 |
| 5.Data collection and audit | 133 | 8.0 (6.0, 9.0) | 7.5 (5.8, 9.0) | 7.0 (6.0, 8.0) | 8.0 (6.2, 8.0) | 0.9 |
| 6.Lack of administrative support | 132 | 8.0 (6.0, 9.0) | 8.0 (6.0, 9.0) | 8.0 (6.0, 9.0) | 8.0 (7.0, 9.0) | >0.9 |
| 7.Recruitment of an ERAS-dedicated nurse | 132 | 8.0 (6.0, 9.0) | 8.0 (6.0, 8.8) | 8.0 (5.0, 9.0) | 9.0 (6.5, 9.5) | 0.3 |

*Data are presented as median and (25^th^-75^th^) percentiles. P values are calculated* from Kruskal-Wallis test.

**Supplementary Table 5D: Barriers to implementation and sustainability of peri-PD ERAS® pathways by case-mix (Abbreviations: ERAS – enhanced recovery after surgery; HPB – hepato-pancreato-biliary; PD – pancreatoduodenectomy)**

| **Characteristic** | **N** | **Overall**, N = 140 | **HPB Surgery**,  N = 96 | **General Surgery**,  N = 25 | **Pancreatic Surgery**,  N = 11 | **Others,** N=8 | **p-value** |
| --- | --- | --- | --- | --- | --- | --- | --- |
| 1.Reluctance to change from the healthcare practitioners | 136 | 8.0 (6.0, 9.0) | 8.0 (6.0, 9.0) | 7.0 (7.0, 9.0) | 7.0 (6.2, 8.8) | 7.5 (5.8, 9.2) | >0.9 |
| 2.Low patient’s involvement and motivation | 136 | 6.0 (4.0, 8.0) | 6.0 (5.0, 8.0) | 6.0 (5.0, 8.0) | 8.0 (3.5, 8.0) | 6.5 (4.0, 8.0) | >0.9 |
| 3.Difficulty in collaboration between members of the multidisciplinary team | 134 | 7.0 (6.0, 8.8) | 7.0 (6.0, 8.0) | 8.0 (6.0, 9.5) | 6.0 (6.0, 8.0) | 7.5 (4.5, 8.5) | 0.4 |
| 4.Initial time and money investment | 135 | 7.0 (5.0, 9.0) | 7.0 (5.0, 9.0) | 7.0 (5.0, 9.0) | 6.5 (5.2, 8.5) | 3.5 (2.0, 8.5) | 0.6 |
| 5.Data collection and audit | 133 | 8.0 (6.0, 9.0) | 8.0 (6.0, 9.0) | 7.0 (5.5, 9.0) | 7.0 (4.2, 7.8) | 8.0 (5.8, 8.0) | 0.7 |
| 6.Lack of administrative support | 132 | 8.0 (6.0, 9.0) | 8.0 (6.0, 9.0) | 8.5 (6.0, 9.2) | 7.0 (6.2, 8.0) | 4.0 (2.8, 7.5) | 0.2 |
| 7.Recruitment of an ERAS-dedicated nurse | 132 | 8.0 (6.0, 9.0) | 8.0 (5.2, 9.0) | 8.0 (5.8, 10.0) | 7.0 (6.0, 8.0) | 8.5 (8.0, 9.0) | 0.5 |

*Data are presented as median and (25^th^-75^th^) percentiles. P values are calculated* from Kruskal-Wallis test.

**Supplementary Table 5E: Barriers to implementation and sustainability of peri-PD ERAS® pathways by annual PD volume (Abbreviations: ERAS – enhanced recovery after surgery; PD – pancreatoduodenectomy)**

| **Characteristic** | **N** | **Overall**, N = 140 | **<20**, N = 33 | **20-50**, N = 57 | **>50**, N = 50 | **p-value** |
| --- | --- | --- | --- | --- | --- | --- |
| 1.Reluctance to change from the healthcare practitioners | 136 | 8 (6.0, 9) | 8 (6.0, 10) | 8 (6.2, 9) | 7 (6.0, 9) | 0.4 |
| 2.Low patient’s involvement and motivation | 136 | 6 (4.0, 8) | 7 (5.0, 9) | 6 (4.5, 8) | 7 (4.0, 8) | 0.4 |
| 3.Difficulty in collaboration between members of the multidisciplinary team | 134 | 7 (6.0, 9) | 8 (7.0, 10) | 7 (6.0, 8) | 7 (5.0, 8) | 0.030 |
| 4.Initial time and money investment | 135 | 7 (5.0, 9) | 8 (6.8, 9) | 7 (5.0, 9) | 6 (5.0, 8) | 0.10 |
| 5.Data collection and audit | 133 | 8 (6.0, 9) | 8 (7.0, 10) | 8 (6.0, 9) | 7 (5.0, 8) | 0.01 |
| 6.Lack of administrative support | 132 | 8 (6.0, 9) | 9 (8.0, 10) | 8 (5.2, 9) | 7 (6.0, 8) | <0.001 |
| 7.Recruitment of an ERAS-dedicated nurse | 132 | 8 (6.0, 9) | 9 (8.0, 10) | 8 (5.0, 9) | 7 (5.0, 9) | 0.001 |

*Data are presented as median and (25^th^-75^th^) percentiles. P values are calculated* from Kruskal-Wallis test.

**Supplementary Table *5*F: Barriers to implementation and sustainability of peri-PD ERAS® pathways by unit’s PD patient care routine care practice (Abbreviations: ERAS – enhanced recovery after surgery; PD – pancreatoduodenectomy)**

| **Characteristic** | **N** | **Overall**, N = 140 | **ERAS® Pathways**, N = 45 | **Post-PD CP to Enhance Recovery**, N = 53 | **Management at Surgeon’s Discretion**, N = 42 | **p-value** |
| --- | --- | --- | --- | --- | --- | --- |
| 1.Reluctance to change from the healthcare practitioners | 136 | 8.0 (6.0, 9.0) | 7.5 (6.0, 8.8) | 7.0 (6.0, 9.0) | 8.0 (7.0, 10.0) | 0.094 |
| 2.Low patient’s involvement and motivation | 136 | 6.0 (4.0, 8.0) | 6.5 (5.0, 8.0) | 6.0 (4.0, 8.0) | 5.5 (4.0, 8.0) | 0.5 |
| 3.Difficulty in collaboration between members of the multidisciplinary team | 134 | 7.0 (6.0, 8.8) | 7.0 (6.0, 8.0) | 7.0 (5.0, 8.0) | 8.0 (6.5, 9.0) | 0.12 |
| 4.Initial time and money investment | 135 | 7.0 (5.0, 9.0) | 7.0 (5.0, 8.5) | 7.0 (4.0, 9.0) | 8.0 (5.8, 9.0) | 0.3 |
| 5.Data collection and audit | 133 | 8.0 (6.0, 9.0) | 8.0 (6.0, 9.0) | 7.0 (5.0, 8.0) | 8.0 (7.0, 9.0) | 0.043 |
| 6.Lack of administrative support | 132 | 8.0 (6.0, 9.0) | 8.0 (6.0, 9.0) | 7.0 (6.0, 9.0) | 8.0 (6.0, 10.0) | 0.6 |
| 7.Recruitment of an ERAS-dedicated nurse | 132 | 8.0 (6.0, 9.0) | 8.0 (5.0, 9.0) | 8.0 (5.5, 9.0) | 8.0 (7.0, 10.0) | 0.4 |

*Data are presented as median and (25^th^-75^th^) percentiles. P values are calculated* from Kruskal-Wallis test.
