# Supplementary material for: Global Perceptions on ERAS® in Pancreatoduodenectomy
Source: World J Surg. 2023 Oct 3;47(12):2977–89. doi: 10.1007/s00268-023-07198-9 (PMC10694106; doi:10.1007/s00268-023-07198-9)
Supplement: Supplementary file 2 — (DOCX 40 kb) [file 268_2023_7198_MOESM2_ESM.docx]

**ERAS® in Pancreatoduodenectomy – An International Online Study (*EPSILON*)**

***Annexure***

**ERAS® in Pancreatoduodenectomy – An International Online Study (*EPSILON*)**

**Survey Questionnaire**

1. **Name & Email (optional):**
2. **Age:**
3. <30years
4. 30-45years
5. 46-60years
6. >60years
7. **Sex:**
8. Female
9. Male
10. Prefer not to say
11. **Practice Location:**
12. Africa
13. America (Central & North)
14. America (South)
15. Asia
16. Europe
17. Oceania
18. **Type of healthcare practice (please select as many as appropriate):**
19. Tertiary hospital
20. Academic / Teaching hospital
21. Regional hospital
22. Public / Government Hospital
23. Private / Corporate hospital
24. Other (please specify): ________________________
25. **Years in clinical practice since obtaining qualifications:**
26. <10years
27. 10-20years
28. 20-30years
29. >30years
30. Retired
31. **Specialty:**
32. Anaesthesia
33. Intensive Care
34. Gastroenterology
35. Nursing
36. Surgery
37. Other (please specify): ________________________
38. **Case-mix of patients treated**
39. Pancreatic surgery unit
40. HPB Unit
41. General surgery
42. Other (please specify): ________________________
43. **What is your Unit’s annual pancreatoduodenectomy volume?**
44. < 20
45. 20-50
46. ≥ 50
47. **What is your Unit’s patient care routine practice?**
48. ERAS®
49. Post-pancreatoduodenectomy clinical pathway to enhance recovery
50. Management at surgeon’s discretion
51. **Years of experience with enhanced recovery practice?**

Specify number of years: ........

1. **How often do you systematically audit pancreatoduodenectomy care processes and outcomes?**
2. Monthly
3. Quarterly
4. Biannually
5. Annually
6. Never
7. Other (please specify): ________________________
8. **Which specialty members take part in the systematic audit meeting?**
9. Anaesthesia
10. Intensive Care
11. Gastroenterology
12. Nursing
13. Surgery
14. Physiotherapist
15. Administrators
16. Other (please specify): ________________________
17. **Which audit system are you using to monitor your perioperative processes?**
18. ERAS Audit system
19. None
20. Other (please specify): ________________________
21. **What do you perceive as the benefits of implementing enhanced recovery in patients undergoing pancreatoduodenectomy? (scale 0 to 10;** (scale 0 = not important, 10 = most important)
22. To reduce length of stay
23. To reduce overall complications
24. To reduce pancreatic surgery-specific complications (POPF, DGE, PPH, PPAP)^1-4^
25. To reduce medical complications
26. To reduce post-pancreatectomy mortality
27. To improves patient satisfaction
28. To decrease the costs
29. To improve oncological outcome
30. None of the above
31. **What is the importance of the following components of ERAS®^5^/Clinical pathways in pancreatoduodenectomy? (scale 0 to 10;** (scale 0 = not important, 10 = most important)
32. Preoperative counselling
33. Pre-habilitation
34. Routine pre-operative biliary drainage
35. Smoking cessation
36. Pre-operative nutritional intervention
37. Carbohydrate loading
38. Pre-anaesthetic medication
39. Thrombotic prophylaxis
40. Antimicrobial prophylaxis and skin preparation
41. Multimodal analgesia
42. Postoperative nausea and vomiting (PONV) prophylaxis
43. Avoiding hypothermia
44. Postoperative glycaemic control
45. Nasogastric (NG) removal at the end of the operation
46. Fluid balance
47. Early removal of Peri-anastomotic drainage
48. Use of somatostatin analogues
49. Urinary drainage
50. Stimulation of bowel movement
51. Early operative nutrition
52. Post-operative mobilization
53. Minimally invasive PD
54. **How challenging would you rate the application of the following components of ERAS®^5^/Clinical pathways in pancreatoduodenectomy? scale 0 to 10;** (scale 0 = not challenging, 10 = most challenging)
55. Preoperative counselling
56. Pre-habilitation
57. Routine pre-operative biliary drainage
58. Smoking cessation
59. Pre-operative nutritional intervention
60. Carbohydrate loading
61. Pre-anaesthetic medication
62. Thrombotic prophylaxis
63. Antimicrobial prophylaxis
64. Analgesia
65. Postoperative nausea and vomiting (PONV) prophylaxis
66. Avoiding hypothermia
67. Postoperative glycaemic control
68. Nasogastric (NG) intubation
69. Fluid balance
70. Peri-anastomotic drainage
71. Use of somatostatin analogues
72. Urinary drainage
73. Stimulation of bowel movement
74. Post-operative nutrition
75. Post-operative mobilization
76. Minimally invasive PD
77. **What are the main facilitators helping to implement and sustain an enhanced recovery pathway?** (scale 0 = not important, 10 = most important)
78. Multidisciplinary and coordination between the different members
79. Patient’s empowerment
80. Regular audit and continuous improvement process
81. ERAS dedicated nurse
82. Clear discharge criteria
83. **What are the main barriers preventing implementation and sustenance of an enhanced recovery pathway following pancreatoduodenectomy?** (scale 0 = not important, 10 = most important)
84. Reluctance to change from the healthcare practitioners
85. Low patient’s involvement and motivation
86. Difficulty in collaboration between members of the multidisciplinary team
87. Initial time and money investment
88. Data collection and audit
89. Lack of administrative support
90. Recruitment of an ERAS-dedicated nurse
91. **Would you be willing to participate in the subsequent rounds of the survey?**
92. Yes
93. No

REFERENCES

1. Wente MN, Bassi C, Dervenis C, et al. Delayed gastric emptying (DGE) after pancreatic surgery: a suggested definition by the International Study Group of Pancreatic Surgery (ISGPS). *Surgery* 2007;142(5):761-8. doi: S0039-6060(07)00301-7 [pii]

10.1016/j.surg.2007.05.005 [published Online First: 2007/11/06]

2. Wente MN, Veit JA, Bassi C, et al. Postpancreatectomy hemorrhage (PPH): an International Study Group of Pancreatic Surgery (ISGPS) definition. *Surgery* 2007;142(1):20-5. doi: S0039-6060(07)00105-5 [pii]

10.1016/j.surg.2007.02.001 [published Online First: 2007/07/17]

3. Bassi C, Marchegiani G, Dervenis C, et al. The 2016 update of the International Study Group (ISGPS) definition and grading of postoperative pancreatic fistula: 11 Years After. *Surgery* 2017;161(3):584-91. doi: S0039-6060(16)30757-7 [pii]

10.1016/j.surg.2016.11.014 [published Online First: 2017/01/04]

4. Marchegiani G, Barreto SG, Bannone E, et al. Postpancreatectomy Acute Pancreatitis (PPAP): Definition and Grading from the International Study Group for Pancreatic Surgery (ISGPS). *Ann Surg* 2021 doi: 10.1097/SLA.0000000000005226 [published Online First: 2021/10/02]

5. Melloul E, Lassen K, Roulin D, et al. Guidelines for Perioperative Care for Pancreatoduodenectomy: Enhanced Recovery After Surgery (ERAS) Recommendations 2019. *World J Surg* 2020;44(7):2056-84. doi: 10.1007/s00268-020-05462-w [published Online First: 2020/03/13]
